# Network changes in patients with phobic postural vertigo

**DOI:** 10.1002/brb3.1622

**Published:** 2020-04-18

**Authors:** Judita Huber, Virginia L. Flanagin, Pauline Popp, Peter zu Eulenburg, Marianne Dieterich

**Affiliations:** ^1^ Graduate School of Systemic Neurosciences Department Biology II Neurobiology Ludwig‐Maximilians‐Universität München Planegg‐Martinsried Germany; ^2^ Research Training Grant 2175 Department Biology II LMU Munich Ludwig‐Maximilians‐Universität München Planegg‐Martinsried Germany; ^3^ German Center for Vertigo and Balance Disorders Ludwig‐Maximilians‐Universität München Klinikum Großhadern München Germany; ^4^ Neurologische Klinik und Poliklinik (Department of Neurology) Ludwig‐Maximilians‐Universität München Klinikum Großhadern München Germany; ^5^ Munich Cluster of Systems Neurology (SyNergy) München Germany

**Keywords:** connectome, dizziness, functional magnetic resonance imaging, vertigo, visual stimulation

## Abstract

**Introduction:**

Functional dizziness comprises a class of dizziness disorders, including phobic postural vertigo (PPV), that cause vestibular symptoms in the absence of a structural organic origin. For this reason, functional brain mechanisms have been implicated in these disorders.

**Methods:**

Here, functional network organization was investigated in 17 PPV patients and 18 healthy controls (HCs) during functional magnetic resonance imaging with a visual motion stimulus, data initially collected and described by Popp et al. (2018). Graph theoretical measures (degree centrality [DC], clustering coefficient [CC], and eccentricity) of 160 nodes within six functional networks were compared between HC and PPV patients during visual motion and static visual patterns.

**Results:**

Graph theoretical measures analyzed during the static condition revealed significantly different DC in the default‐mode, sensorimotor, and cerebellar networks. Furthermore, significantly different group differences in network organization changes between static visual and visual motion stimulation were observed. In PPV, DC and CC showed a significantly stronger increase in the sensorimotor network during visual stimulation, whereas cerebellar network showed a significantly stronger decrease in DC.

**Conclusion:**

These results suggest that the altered visual motion processing seen in PPV patients may arise from a modified state of sensory and cerebellar network connectivity.

## Graphical abstract

Individuals with phobic postural vertigo (PPV) display vestibular symptoms in the absence of a structural or organic origin. For this reason, functional brain mechanisms have been implicated in these disorders. Here, functional network organisation was investigated in PPV patients during functional magnetic resonance imaging with a visual motion stimulus. Notable network organisation changes were found in the sensorimotor and the cerebellar networks.

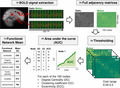

**Abbreviations**
AUC: area under the curve
CC: clustering coefficient
DC: degree centrality
ECC: eccentricity
FD: framewise displacement
fMRI: functional magnetic resonance imaging
HC: healthy controls
PPPD: persistent postural‐perceptual dizziness
PPV: phobic postural vertigo
ROI: region of interest
rs‐fMRI: resting‐state functional magnetic resonance imaging

## 1 INTRODUCTION

One of the most common diagnoses in neuro‐otology centers is functional dizziness with an estimated prevalence of 10% (Dieterich & Staab, 2017). Functional dizziness, previously known as somatoform or psychogenic dizziness, refers to a class of chronic dizziness disorders with a highly overlapping etiology (Dieterich & Staab, 2017). Although the disorder may be precipitated by a structural vestibular syndrome, the chronic manifestation of vertigo, dizziness, or unsteadiness symptoms has no structural origin. Key symptoms include persistent dizziness and unsteadiness that is usually exacerbated by upright posture, motion, or visual motion stimulation (Dieterich & Staab, 2017). Furthermore, functional dizziness often co‐occurs with obsessive‐compulsive personality traits and symptoms of anxiety and depression (Brandt, 1996; Staab et al., 2017).

Functional dizziness includes phobic postural vertigo (PPV) (Brandt, 1996), chronic subjective dizziness (Ruckenstein & Staab, 2009), visually induced dizziness (Bisdorff, Von Brevern, Lempert, & Newman‐Toker, 2009; Bronstein, 1995), and space and motion discomfort (Jacob, Lilienfeld, Furman, Durrant, & Turner, 1989). The disorders typically differ in their provocative factors, temporal profile, or the focus of the diagnosis. Recently, the Bárány Society Classification Committee developed diagnostic criteria that incorporate all these dizziness disorders into a common disorder called persistent postural‐perceptual dizziness (PPPD) (Dieterich & Staab, 2017; Popkirov, Staab, & Stone, 2018; Staab et al., 2017). In the current study, patients were recruited before 2017 and thus used the original PPV criteria (Brandt, 1996; Lempert, Brandt, Dieterich, & Huppert, 1991). Patients are therefore referred to as PPV patients here, although they would fall under the new PPPD classification. Because PPPD is a recent classification and less well established in the literature, we use the term functional dizziness to discuss previous literature using patient populations described having chronic subjective dizziness, visually induced dizziness, or space and motion discomfort.

One of the hallmark features of functional dizziness is the task dependency of symptoms such as postural performance. While patients show increased body sway during simple standing tasks, they typically improve during more difficult balance tasks. In contrast, healthy individuals typically worsen with an increasing difficulty in balancing tasks (Holmberg, Tjernström, Karlberg, Fransson, & Magnusson, 2009; Querner, Krafczyk, Dieterich, & Brandt, 2000). Furthermore, when a balance task is combined with a distraction task, PPV patients showed the same amount of body sway and cocontraction of leg antigravity muscles as healthy controls (HCs), that is, their balancing behavior normalizes (Wuehr, Brandt, & Schniepp, 2017). This has led to the idea that functional changes in monitoring, predicting, and attention to bodily perceptions are altered in these patients.

Although the behavioral characteristics of functional dizziness disorders have been identified, their neural attributes are not yet understood. Since evaluation and interpretation of sensory stimuli appear disrupted in functional dizziness patients, information processing is likely affected in sensory brain areas. Furthermore, the cerebellum is often considered as a key structure in predicting perceptual events (Baumann et al., 2015) and as being a control structure for automatic movements (Jahn, Deutschländer, Stephan, Kalla, Hüfner, et al., 2008; Jahn, Deutschländer, Stephan, Kalla, Wiesmann, et al., 2008). Cerebellar activity and connectivity might thus also be related to the dysfunctional behavior in functional dizziness.

Few imaging studies have investigated the neural characteristics of functional dizziness disorders. For example, in the study by Indovina et al. (2015) functional connectivity changes between visual, vestibular, and anxiety‐related brain regions in functional dizziness patients were investigated. They found more negative functional connectivity changes in these regions upon sound‐evoked vestibular stimulation, when compared to HCs. This suggests an altered coordination between sensory and higher cortical regions in these patients (Indovina et al., 2015). Alterations in sensory and cerebellar brain connectivity were found in functional dizziness patients during resting‐state functional magnetic resonance imaging (rs‐fMRI) (Van Ombergen et al., 2017). Another recent rs‐fMRI study differentiating comorbid anxiety and depression from PPPV suggested that increased connectivity in the occipital areas was more related to comorbid disorders, while decreased connectivity among vestibular, frontal regulatory, and visual cortices, as well as decreased connectivity between cerebellar regions, was rather related to functional dizziness itself (Lee et al., 2018). A task‐based fMRI approach using a visual motion aftereffect paradigm to study task‐related activity and task‐based connectivity was performed in PPV patients (Popp et al., 2018). Here, the prefrontal cortex showed increased gray matter volume and increased connectivity with associated thalamic projections and primary motor areas. Conversely, decreased gray matter and connectivity were found in cerebellar vermis, posterior lobules, and the supramarginal gyrus. These results pointed to a higher weighting of cognitive‐based control of motor areas during a sensory task that induced dizziness in PPV patients (Popp et al., 2018).

These results suggest that brain function and connectivity differ in functional dizziness patients, even in the absence of an organic dysfunction. So far, however, no specific region or mechanism has emerged from the studies. Instead, a distributed array of regions appears to be implicated in functional dizziness, pointing toward network differences in these patients. Furthermore, considering that normal posture and gait can occur in these patients under certain conditions immediately after dysfunctional balancing (Querner et al., 2000; Schniepp et al., 2014; Wuehr et al., 2017), network organization may be influenced by differential sensory processing. Therefore, we examined the whole‐brain network architecture during episodes of visual motion, compared to a static visual stimulus. To this aim, we used a graph theoretical approach to extensively analyze the network properties of the whole brain in PPV patients using the data collected in Popp et al. (2018). Six well‐known functional subnetworks were characterized in terms of their importance, segregation, and functional integration of the network (degree centrality [DC], clustering coefficient [CC], and eccentricity [ECC], respectively) (Bullmore & Sporns, 2009; Rubinov & Sporns, 2010). These measures during visual motion stimulation were then compared with those during a static visual stimulation as well as between PPV patients and HCs.

## 2 METHODS

### 2.1 Participants

This study used the data from Popp et al. (2018) to analyze differences in functional connectivity between PPV patients and HC. Overall, 34 patients and 37 HC were included in the original study (Popp et al., 2018). Patients were recruited from the Dizziness Clinic of the University Hospital Munich (German Center for Vertigo and Balance Disorders). The study was approved by the local ethics committee of the Ludwig‐Maximilians‐Universität München, Germany. All subjects gave their informed written consent to participate in the study.

Phobic postural vertigo was diagnosed based upon the criteria by Brandt (1996) as determined after diagnostic testing at the German Center for Vertigo and Balance Disorders (DSGZ) in Munich. Patients presented with (a) persistent nonspinning dizziness or unsteadiness while standing or walking despite normal clinical balance tests; (b) perceptual or social factors typically exacerbate the symptoms leading to conditioning and avoidance behavior; (c) fluctuating unsteadiness from seconds to minutes; (d) frequent onset after a serious illness, a vestibular disorder, or a period of emotional stress; (e) vegetative symptoms or anxiety during or after vertigo; and (f) an obsessive‐compulsive personality type, mild depression, or a labile affect. These symptoms must present either in the absence of a structural origin or as a secondary symptom after an acute but now compensated vestibular pathology. The absence of a structural pathology was determined by a clinical neurological examination and a neuro‐orthoptic examination including video head impulse test (vHIT), caloric irrigation, measurements of subjective visual vertical, posturography, and structural magnetic resonance imaging of the brain.

A high number of patients terminated the experiment early and displayed high head motion, particularly in later sessions of the experiment. Therefore, participants had to complete the first session and had a maximum head motion of 3 mm or maximum head motion of 3 degrees to be included in the analysis so as not to introduce additional variability due to differences in the number of samples for the network analysis (18 patients and 18 HCs). One additional patient had to be excluded for excessive head movements (see Section 2.4.1). We thus ended up with 17 right‐handed patients (8 female) diagnosed with PPV patients and 18 right‐handed HC (7 female) in the current analyses. The mean age of PPV patients was 41.47 years (*SD* = 11.33 years). In HC, the mean age was 36.11 years (*SD* = 12.93 years). Groups did not significantly differ in terms of age (*t*_(32.82)_ = −1.306, *p* = .201), but because of the potentially still relevant difference in mean age between the cohorts, we used age as a relevant covariate in our analysis.

### 2.2 MR parameters

MR data were acquired on a 3T MRI machine (GE, Signa Excite HD), using a 12‐channel head coil. A T2*‐weighted gradient‐echo echo‐planar imaging sequence sensitive to blood‐oxygen‐level‐dependent (BOLD) contrast was used to collect functional images (TR 2.45 s, TE 40 ms, FA 90°, voxel size 3 mm isotropic, 38 transversal slices). Three consecutive functional runs were acquired, each containing 260 volumes covering the whole brain. The total number of volumes did not include the first four volumes, which were not reconstructed because they contain transient T1 effects. Slices were collected in an ascending interleaved fashion. We analyzed the first completed session for each participant. A T1‐weighted anatomical image (FSPGR, slice thickness = 0.7 mm, matrix size = 256 × 256, FOV = 220 mm, phase encoding = anterior/posterior, FA = 15 ms, bandwidth = 31.25, voxel size = 0.86 × 0.86 0.7 mm) was acquired at the start of the MRI session.

### 2.3 Task description

Participants received earplugs in combination with sound‐isolating headphones for a profound noise reduction inside the MRI machine. Our visual stimulus consisted of 600 black and white dots (diameter = 0.5°) randomly positioned on a gray background. The dots moved coherently at a constant speed (7°/sec) for the duration of 27.5 s (herewith called “motion” stimulus). After this time period, static dots were shown for another 27.5 s (herewith called “static” stimulus). Each run was 11 min long with 12 blocks of the motion stimulus. The motion stimulus could move to the left, right, counterclockwise, or clockwise and change from one block to the other. Participants were asked to passively look straight ahead *through* the visual stimulus. Instantly after the end of the motion stimulus, participants had to press a button when they no longer experienced the motion aftereffect (the feeling that the static dots were moving into the opposite direction from the precedent stimulus). MATLAB 8.0 (The MathWorks, Inc., Natick, Massachusetts, US) was used together with the Cogent 2000 toolbox (http://www.vislab.ucl.ac.uk/cogent_2000.php) to present the visual stimuli. The field of view was ±24.9° in the horizontal and ±18.9° in the vertical plane. The visual field was kept small to prevent sensations of vection.

### 2.4 Preprocessing

Image preprocessing was performed using DPARSF (RRID:SCR_002372, version 4.3_170105) toolbox with MATLAB 2016 (RRID:SCR_001622, The MathWorks, Inc.). Functional images of each participant were realigned to the first. The T1 images were segmented using the affine regularization in DARTEL and subsequently coregistered to the mean functional image. Both functional and structural images were normalized using DARTEL into MNI space at a voxel size of 2 mm^3^. Functional images were additionally smoothed during the normalization process using a Gaussian smoothing kernel with FWHM of 4 mm.

#### 2.4.1 Head motion

Head movements may induce spurious correlations of the fMRI time courses with each other (Power, Barnes, Snyder, Schlaggar, & Petersen, 2012) and distort graph measures (Yan, Craddock, He, & Milham, 2013). Therefore, mean motion and correlations of head movement with task on‐ and offsets were inspected and compared between PPV patients and HC. Head motion was determined using framewise displacement (FD) calculated according to Jenkinson (Jenkinson, Bannister, Brady, & Smith, 2002) as implemented within the DPARSF toolbox. This measure was recommended over other head motion parameters by Yan, Cheung, et al. (2013).

For all participants, the following two FD measures were used. First, mean FD was calculated across the whole scanning session (260 time points). Second, the correlation between FD and the task was determined as the Pearson correlation between the binary vector representing task on‐ and offsets and the FD vector across the scanning session. Therefore, we determined not only whether participants moved excessively in general but also to what degree head movement coincided with the task. Values with a normalized *z*‐score of >±3 within each group led to exclusion of the subject's data set.

In the PPV group, one patient had to be excluded due to high mean FD (mean FD = 0.153, *z* = 3.191). No other individual from the PPV group had to be excluded due to excessive task–motion correlation. Within the HC group, no outlier values were found. No HC was therefore excluded from further analysis.

Differences in head motion between groups were analyzed to assure validity of the network analysis. Assumptions for homogeneity of variances were tested for each group using *F* test; assumptions of normality were tested using Shapiro–Wilk normality test. If assumptions of homogeneity and normality were met, two‐sample *t* test was used for group comparison; else, nonparametric Wilcoxon rank test was used.

Nonparametric tests were used to determine differences of mean FD between groups; groups did not differ significantly in mean FD (*W* = 124, *p* = .351) (Figure 2c). Group differences between task–movement correlations were tested using a parametric two‐sample *t* test since all necessary assumptions were met. Indeed, group differences were found (*t*_(33)_ = −2.203, *p* = .035) with correlation of motion with task onsets being significantly higher in PPV patients compared to HC (Figure 2d). To take this into consideration, we removed motion parameters from the original BOLD signal, as described in the following section.

#### 2.4.2 Data extraction and cleaning

**FIGURE 1:**
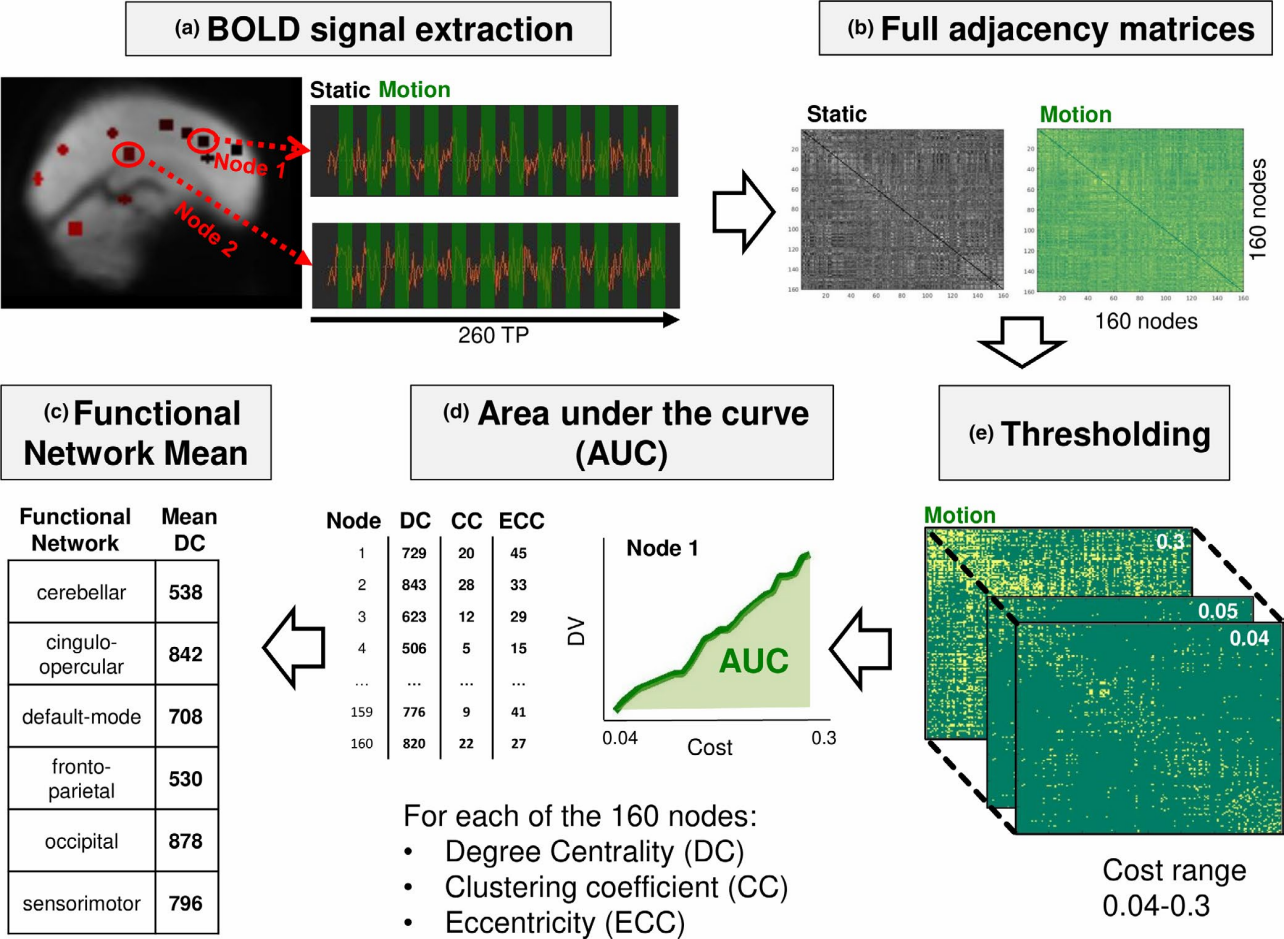
The analysis pipeline used in this study, shown for one example participant. The analysis pipeline was loosely based on previous analysis approaches (e.g., Bassett et al. (2012) and Markett, Montag, Melchers, Weber, and Reuter (2016). (a) The BOLD signal was extracted from 160 Dosenbach nodes for all 260 time points. Signal includes periods where participants were shown a visual motion stimulus (“motion”), interspersed with periods with a static visual stimulus (“static”). (b) Adjacency matrices for each participant were created for the static and motion condition by using hemodynamic response function weighting and bivariate correlation. (c) Binarized matrices were created with a range of costs (0.04–0.3, steps of 0.01), which was determined as being the thresholds where small‐world dynamics were preserved. (d) For each threshold, three measures were calculated: degree centrality, clustering coefficient, and eccentricity. Area under the curve (AUC) was calculated for each node and each graph measure. (e) Mean over nodes belonging to the same network, for each of the graph measures. Here, only values for mean DC are shown; however, they were calculated for clustering coefficient and shortest path as well. DC, degree centrality

Subsequent processing was performed using the CONN toolbox (RRID:SCR_009550, version 17.f) (Whitfield‐Gabrieli & Nieto‐Castanon, 2012). For each participant, inputs to the CONN processing pipeline included the preprocessed functional and structural images, as well as the normalized gray matter, white matter (WM), and cerebrospinal fluid (CSF) masks. The mean BOLD signal was extracted from 160 region of interest (ROIs) (4.5‐mm‐radius spheres), according to the Dosenbach atlas (Dosenbach et al., 2010) (Figure 1a). The atlas was downloaded from ABIDE Open Connectomes Project website (http://preprocessed‐connectomes‐project.org/abide/Pipelines.html). Six motion parameters (three rotation and three translation parameters) were entered as first‐level covariates, and group identity vectors (patients and controls) were entered as second‐level parameters. A principal component analysis (PCA) was performed to determine the signals explaining the most variance in the WM and CSF.

The time series were then denoised. First, the first five principal components from the PCA and the 6 motion parameters were removed via linear regression. Because we were interested in functional connectivity which cannot be explained by task‐specific co‐activations, the time series convolved with the hemodynamic response function for the task effects of the “motion” and “static” conditions were also regressed out of the BOLD signals. After regression, data were high‐pass‐filtered with a cutoff of 0.008 Hz to remove any scanner‐related drifts in the signal. No low‐pass filter was applied to avoid possible signal spillage of the BOLD signal between different conditions and to avoid filtering out possible task signals at higher frequencies (Cole, Bassett, Power, Braver, & Petersen, 2014). Finally, the time series were detrended and despiked, as implemented in the CONN toolbox. The resulting BOLD signals from the 160 regions were used for data analysis.

### 2.5 Data analysis

Graph theory was used to characterize brain network connectivity. In this method, the brain is defined as a set of nodes connected to each other via edges, thus forming a graph (Fornito, Zalesky, & Bullmore, 2016). In the context of fMRI, edges are derived from the Pearson correlation between BOLD signal time courses of the two respective nodes (Fornito et al., 2016). In the following, the analysis steps will be specified (also see Figure 1 for a graphical representation).

#### 2.5.1 Adjacency matrix

We were interested in investigating potential differences in connectivity separately during static and motion conditions. To achieve this, we used the standard approach implemented in CONN to determine “condition‐dependent” functional connectivity. Specifically, a weighted GLM was performed to determine the BOLD signals specific for the static and the motion conditions, respectively. For this, the block regressors are convolved with the hemodynamic response function, thus creating a measure of how each scan is expected to be affected by each task. This regressor is then further used to weight each scan in order to compute a weighted correlation across all time points (also see Whitfield‐Gabrieli & Nieto‐Castanon, 2012). The correlations computed for each ROI were included in two 160x160 adjacency matrices for each participant, one for each condition (static and motion) with the correlation value between all nodes described as a *z*‐score (Figure 1b). Note that anticorrelations were not considered for the analysis; therefore, only positive z‐scores were used for the subsequent calculations.

#### 2.5.2 Graph measures

Three graph measures were chosen to describe network properties: DC, CC, and ECC. DC is the total number of edges that connect the node to the remaining network (Bullmore & Sporns, 2009). A node with a high DC will interact highly with the remaining nodes of the network (Fornito et al., 2016; Rubinov & Sporns, 2010). CC measures the number of pairs of a node's neighbors that are connected with each other as a fraction of the total amount of pairs that particular node has (Fornito et al., 2016). Paths in a network are a distinct sequence of a route of information flow. ECC is a nodal measure for path length and is defined as the maximum shortest path length between a node and any other node, thus describing how functionally integrated a node is (Rubinov & Sporns, 2010).

#### 2.5.3 Thresholding

In order to calculate graph theory measures from the adjacency matrices, thresholding is usually performed to remove spurious links with low correlation values (Fornito et al., 2016). It has been suggested that density thresholding is more appropriate than absolute thresholding to keep the number of links in the graph stable. This way, possible differences in graph properties do not merely emerge due to different connection density. Relative thresholding is thus particularly suited for comparing brain graphs between groups of participants (De Vico Fallani, Richiardi, Chavez, & Achard, 2014; Fornito et al., 2016). However, often only one arbitrary proportional threshold (or “network cost”) is chosen for a network which might also lead to erroneous results.

**FIGURE 2:**
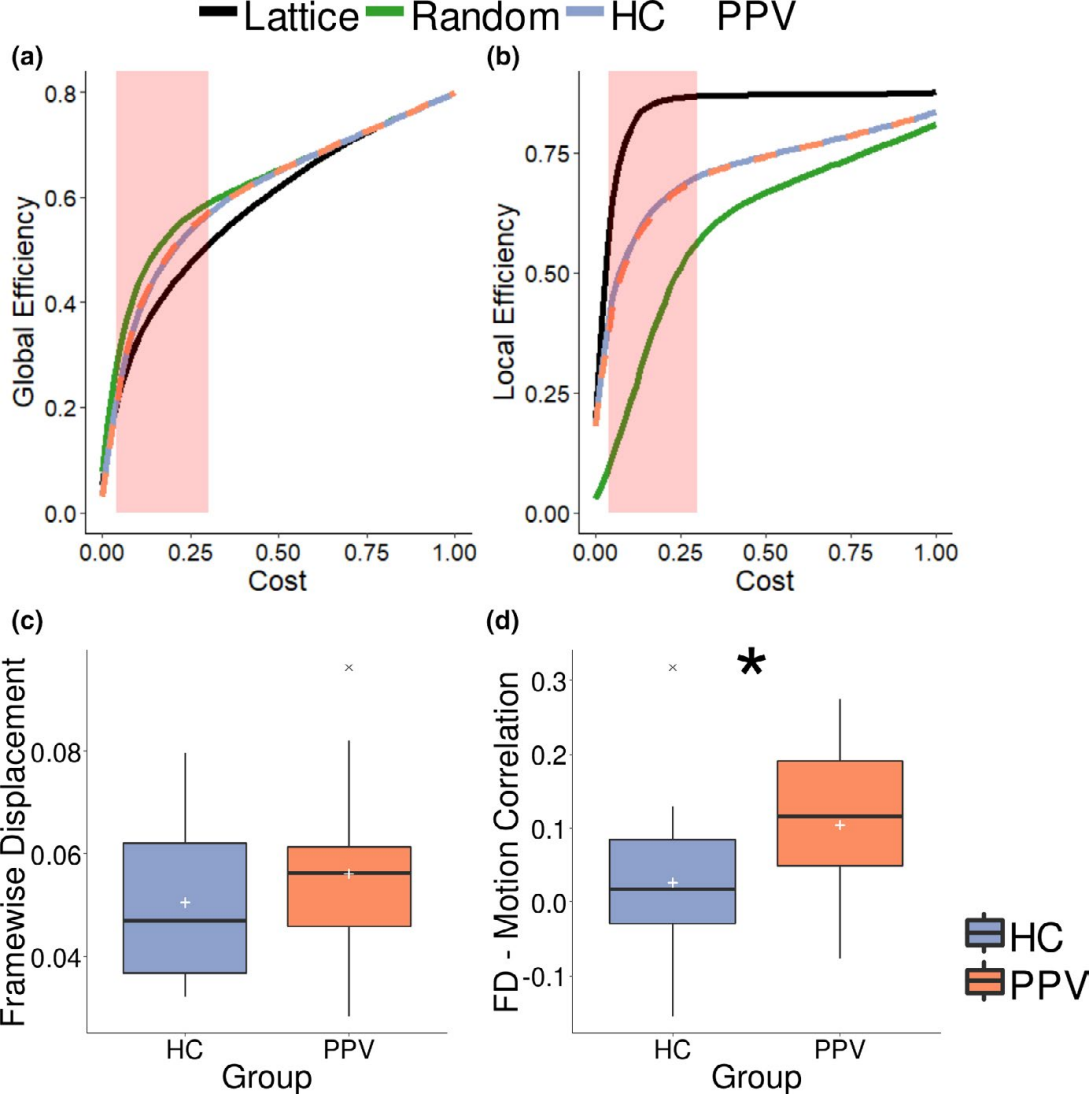
(a) Global efficiency for real graphs (healthy controls [HCs] and patients [PPV]) and shuffled graphs (random and lattice) at different costs. Small‐world regime occurs between thresholds of 0.04 and 0.3 (highlighted in red). (b) Local efficiency for real graphs (HC and PPV patients) and shuffled graphs (random and lattice) at different costs. (c) Box plot showing mean framewise displacement (FD) over the course of the whole session for HC and PPV patients (white cross indicates mean). (d) Box plot showing correlation between FD and a vector modeling onset and offset of visual stimulation for HC and PPV patients (white cross indicates mean). PPV, phobic postural vertigo

We therefore adopted the approach of calculating graph measures over a range of threshold values (similar to Bassett, Nelson, Mueller, Camchong, & Lim, 2012; Ginestet, Nichols, Bullmore, & Simmons, 2011) instead of choosing one arbitrary network cost. The range of threshold values was chosen such that networks had small‐world properties, as would be expected from a biologically plausible network (Achard & Bullmore, 2007). A small‐world network should have a global efficiency greater than a lattice graph but smaller than a random graph (Achard & Bullmore, 2007). Furthermore, local efficiency of a small‐world network should be lower than a lattice graph and higher than a random graph. For this, global and local efficiency of all participants during static periods were compared with global and local efficiency of randomized and lattice graphs. Using the *randmio_und* and *latmio_und* functions of the Brain Connectivity Toolbox (Rubinov & Sporns, 2010, RRID:SCR_004841, version from 15.01.2017), the graph of each participant was both permuted to a random and a lattice graph for costs in the interval of 0.01–0.60 using a step size of 0.02 and a rewiring parameter of 100. Global and local efficiency were calculated for each cost (Figure 2a,b). Small‐world properties were found in the range of costs between 0.04 and 0.3 (Figure 2a), similar to Achard and Bullmore (2007). This cost range was used for all subsequent calculations (Figure 1c).

For each thresholded matrix (0.04–0.3, steps of 0.01), adjacency matrices were binarized using the functions *threshold_proportional* and *weight_conversion* from the Brain Connectivity Toolbox (Figure 1c). For ECC, a distance matrix was calculated using the function distance_bin. DC, CC, and ECC were calculated using functions for undirected binary networks from the Brain Connectivity toolbox, respectively. Therefore, each node could be described with three graph measures calculated using 35 different thresholds.

To summarize these values, for each of the 160 atlas nodes and for each graph measure, the area under the curve (AUC) was calculated, resulting in 160 × 3 values for each participant (Figure 1d). Since we were mainly interested in characterizing network properties of functional networks, we grouped every node into one of six networks: cingulo‐opercular, fronto‐parietal, default‐mode, sensorimotor, occipital, and cerebellum (Figure 1e) (according to Dosenbach et al., 2010). For each network, we thus calculated the mean AUC from the respective nodes. Therefore, in the end, each participant had 18 summary network measures for each condition: the AUC for the three graph measures for the six networks. These were calculated for both static and motion periods, thus resulting in 36 measures overall for each participant.

#### 2.5.4 Group statistics

We first tested for differences between network properties in each stimulation condition separately, and then by subtracting the summary graph measures of the static condition from the motion condition (motion–static). In both cases, we used a mixed‐design ANCOVA with “group” as between‐group factor, “network” as within‐group factor, and age of participants as a covariate. In case of a significant Mauchly test of sphericity, Greenhouse–Geisser correction for departure from sphericity was reported. We were interested in differences between groups, rather than differences solely explained by the heterogeneity of networks across groups. Therefore, only in the case of significant main effects of “group” or an interaction of “group” with “network,” post hoc pairwise *t* tests were used to determine the nature of the difference using FWE correction using Tukey's method. All calculations were performed using lsmeans (Lenth, 2016), afex (Singmann, Bolker, Westfall, & Aust, 2018), plyr (Wickham, 2011), and reshape (Wickham, 2007) libraries in R 3.4.0 (RRID:SCR_001905, 2018). Human brain networks were visualized using BrainNet Viewer (Xia, Wang, & He, 2013, RRID:SCR_009446). All analysis and plotting of results were performed using R 3.4.0, Python 3, and MATLAB 2016 (The MathWorks, Inc.).

## 3 RESULTS

### 3.1 Connectivity group effects during static and motion condition

To test for the presence of general differences in any of the measures, we performed a MANCOVA to determine the overall group, network, or interaction effect on any graph measure during static and motion conditions, as well as the effect of age. By including the factor “network” as a repeated‐measure factor and “group” as an independent‐measure factor, we aimed to minimize unexplained variance from the model*.* Three graph measures (DC, CC, and ECC) were included as dependent variables, and group of participants and six functional networks were included as independent variables. Age was added as a covariate. Note that the main significant results below remain, even if we correct our initial significant p‐value for multiple testing using Bonferroni correction since three MANCOVAs were tested (i.e., if we adjust the criterion to *p* = .0167).

To additionally investigate effects of motion, an alternative model was tested that included a subject‐specific nuisance regressor for regressing out the signal related to time points with excessive motion (see Supplementary Information, Analysis 1). We also conducted the same analysis with normalized values by the estimated values for a random graph (see Supplementary Information, Analysis 2). Unless otherwise stated, the results in these alternative analyses yielded the same results.

**FIGURE 3:**
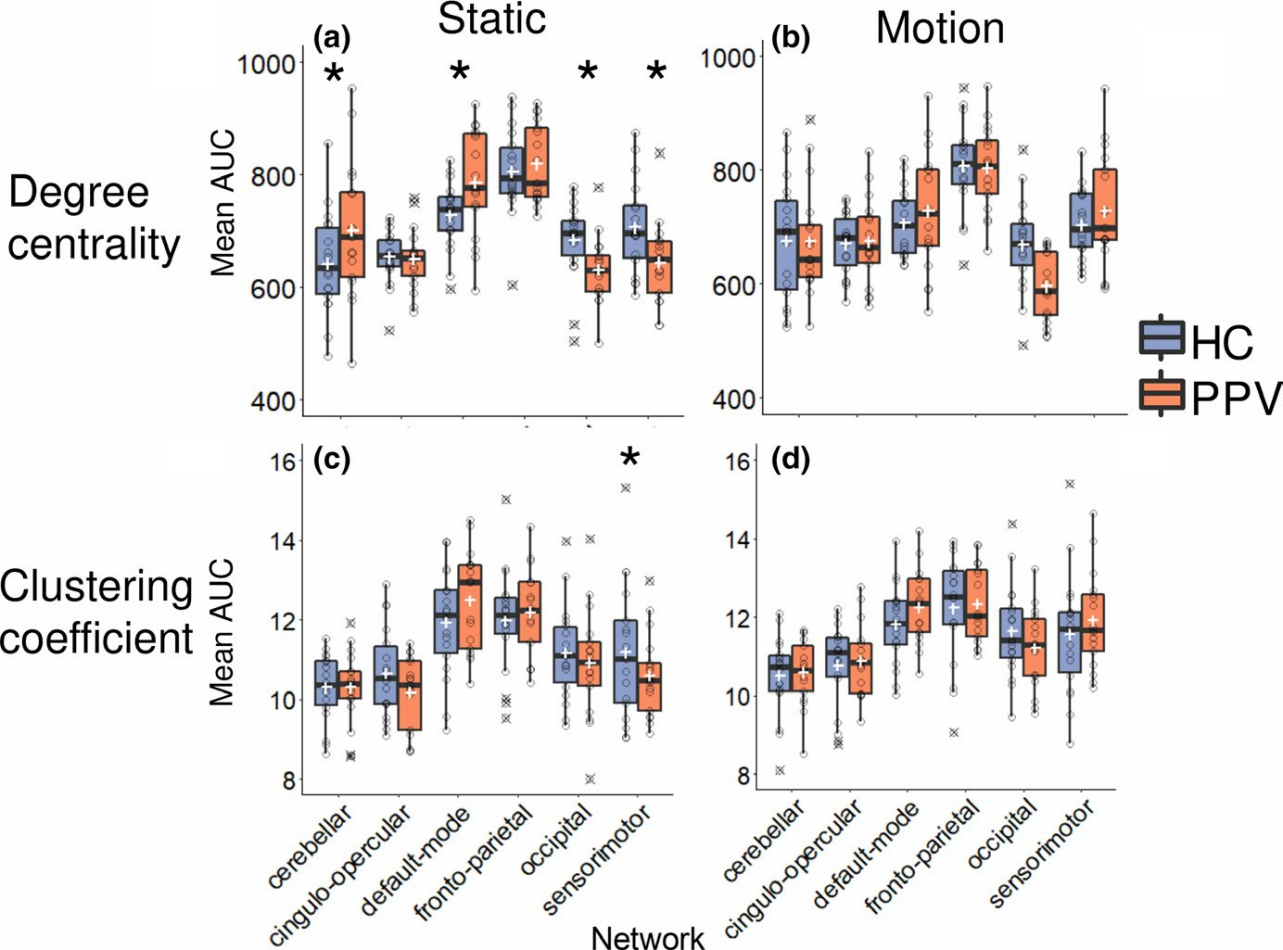
Box plots comparing degree centrality mean area under the curve (AUC) and clustering coefficient AUC between healthy controls (HCs) and patients (PPV) both in static and in motion, for each of the six functional brain networks given by Dosenbach (2010). White crosses indicate means, stars indicate a significant (*p* < .05) group effect, and outliers are marked with a black cross. (a) Degree centrality during static conditions. (b) Degree centrality during motion conditions. (c) Clustering coefficient during static conditions. (d) Clustering coefficient during visual motion conditions. PPV, phobic postural vertigo

During the visual motion condition, no significant interaction (Pillai's trace = 0.062 *F*_(5,15)_ = 0.658, *p* = .826) or main group effects were found (Pillai's trace = 0.087, *F*_(3,29)_ = 0.924, *p* = .441). The main effect of age (Pillai's trace = 0.370, *F*_(3,29)_ = 5.681, *p* = .003), as well as the factor of network, was found to be significant using MANCOVA (Pillai's trace = 0.844, *F*_(5,15)_ = 12.143, *p* < .001). No subsequent ANCOVAs were thus performed for this condition (Figure 3b,d).

For the static condition, however, the interaction between group and network effects was significant (Pillai's trace = 0.187, *F*_(15,465)_ = 2.057, *p* = .011). There was no significant main effect of group (Pillai's trace = 0.023, *F*_(3,29)_ = 0.234, *p* = .871) and no main effect of age (Pillai's trace = 0.159, *F*_(3,29)_ = 1.834, *p* = .163). Furthermore, a significant main effect of the factor network was found (Pillai's trace = 0.740, *F*_(15,465)_ = 10.145, *p* < .001).Consequently, a subsequent post hoc analysis was used to determine what network properties show differences between PPV patients and HC in specific networks. For this, three separate mixed‐design ANCOVAs were performed, one for each of the network measures, DC, CC, and ECC during the static condition.

For DC, the factor group and network showed a significant interaction (*F*_(3.41,109.12)_ = 3.266, *p* = .019, where degrees of freedom were adjusted using Greenhouse–Geisser estimates of sphericity (*ε* = 0.683) after Mauchly's test indicated that the assumption of sphericity had been violated [*W*_(14)_=0.355, *p* = .005]). Both the factor of group (*F*_(1,32)_=0.062, *p* = .435) and the main effect of age (*F*_(1,32)_=0.136, *p* = .715) were not found significant. The main effect of network was found significant (*F*_(3.41,109.12)_=20.068, *p* < .001 after adjusting degrees of freedom as above). Because of the significant interaction, post hoc *t* tests were performed using Tukey's method to test in which networks the group effect was most pronounced. Indeed, DC of cerebellar network nodes (*t*_(168.32)_ = −2.245, *p* = .0260) and default‐mode network nodes (*t*_(168.32)_ = −2.201, *p* = .0291) was higher in PPV patients compared to HC (Figure 3a). In contrast, DC of sensorimotor nodes (*t*_(168.32)_ = 2.389, *p* = .018) was lower in PPV patients when compared to HC (Figure 3a). PPV patients also had a lower DC of occipital nodes, compared to HC (*t*_(168.32)_ = 1.996, *p* = .048), but this result did not survive in the model for subject‐specific motion (see Analysis 1 in Supplementary Information). Individual within‐participant changes in DC can be seen in Figure A.1.

For CC, a significant interaction between the factor of group and network was also found (*F*_(3.57,114.24)_ = 2.560, *p* = .046). Degrees of freedom were adjusted using Greenhouse–Geisser estimates of sphericity (*ε* = 0.714), since Mauchly's test indicated that the assumption of sphericity was violated, *W*_(14)_ = 0.385, *p* = .012. No main effect of group was found (*F*_(1,32)_ = 0.219, *p* = .643). However, the main factor of age was found to be significant (*F*_(1,32)_ = 0.029, *p* = .029). The main effect of network was also significant (*F*_(3.57,114.24)_ = 26.817, *p* < .001), degrees of freedom were adjusted as above). Because of the significant interaction, we performed post hoc *t* tests using Tukey's method to determine in which networks CC significantly differed between HC and PPV patients. The only significant effect was found in the sensorimotor network (*t*_(95.73)_ = 2.014, *p* = .047); HC showed a higher CC in the sensorimotor network (Figure 3c) than PPV patients. For an overview of within‐participant changes in CC, see Figure A.2.

For ECC, no significant main effect or interaction was found during the static condition (Figure A.3a). Within‐participant ECC values for each network can be seen in Figure A.4.

### 3.2 Change of graph measures between conditions

We were further interested in the relative change in network properties between the visual motion and static visual conditions. For this, for each participant and graph measure, the values of each node during the static condition were subtracted from the motion condition, thus resulting in values representing the change of degree centrality (ΔDC), clustering coefficient (ΔCC), and eccentricity (ΔECC). This resulting value indicates whether the mean AUC for one graph measure of a certain network remained the same between conditions (and thus has a value close to zero), or whether it increased during motion (positive) or decreased during motion (negative).

An initial MANCOVA resulted in a significant interaction between the factors group and network (Pillai's trace = 0.176, *F*_(15,465)_ = 1.933, *p* = .019), as well as a main effect of group (Pillai's trace = 0.313, *F*_(3,29)_ = 4.409, *p* = .0113) and a main effect of network (Pillai's trace = 0.182, *F*_(15,465)_ = 2.008, *p* = .0135). The covariate of age was not significant (Pillai's trace = 0.066, *F*_(3,29)_ = 0.679, *p* = .572). As before, the specific effects for each graph measure was determined via mixed‐design ANCOVAs for ΔDC, ΔCC, and ΔECC. Only ΔDC and ΔCC showed significant differences between HC and PPV patients. For ΔECC, no significant interaction or main group effect was found (Figure A.5). For ΔDC, a significant interaction was found between the factor of group and network (*F*_(3.97, 127.04)_ = 3.456, *p* = .010). Degrees of freedom were adjusted using Greenhouse–Geisser estimates of sphericity (*ε* = 0.794) after Mauchly's test indicated that the assumption of sphericity was violated (*W*_(14)_ = 0.422, *p* = .027). A significant main effect of network (*F*_(3.97, 127.04)_ = 4.477, *p* = .002 degrees of freedom were adjusted as described above) and group (*F*_(1,32)_ = 7.096, *p* = .012) was also found. No significant main effect of age was found (*F*_(1,32)_ = 0.017, *p* = .897).

**FIGURE 4:**
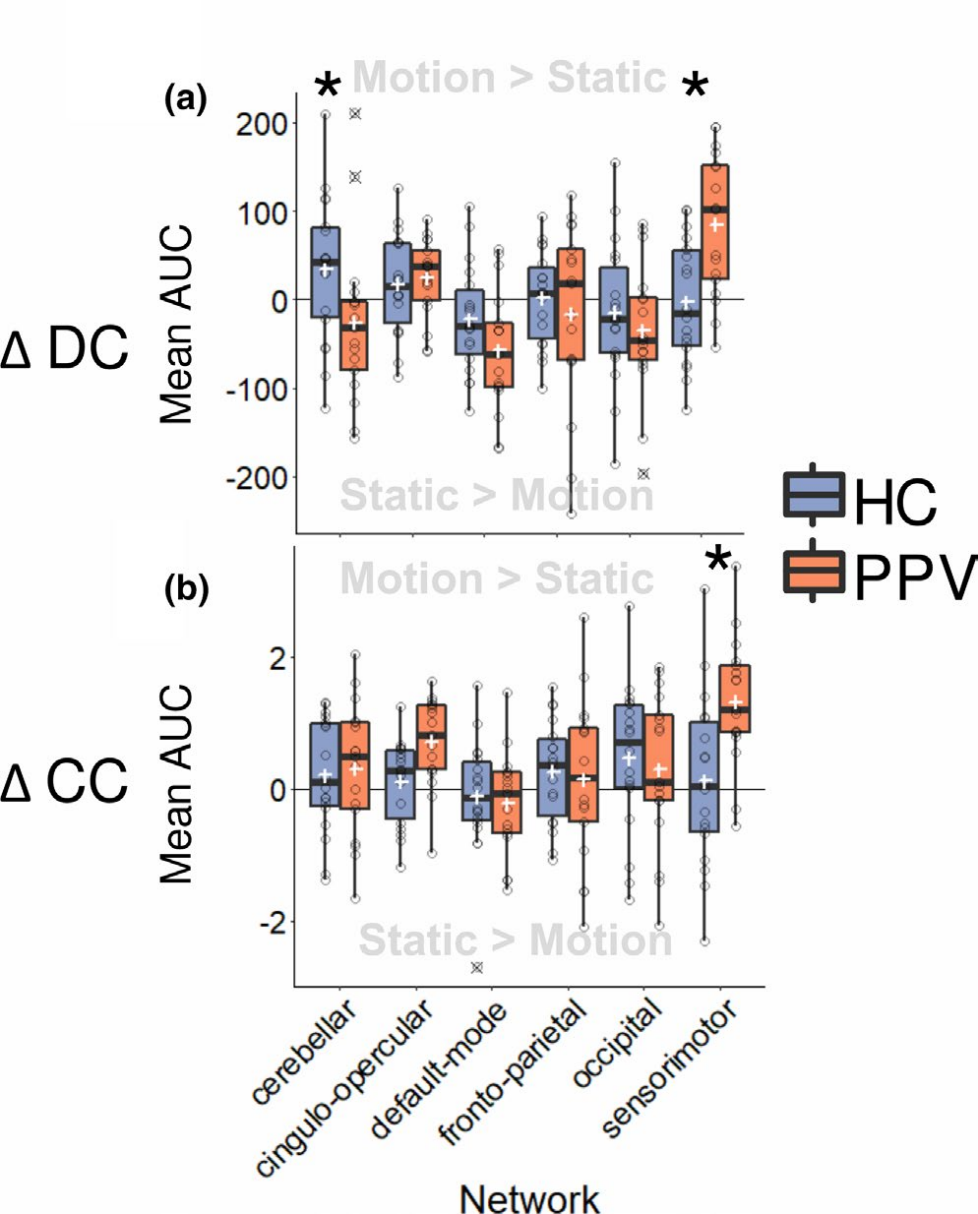
Box plots showing (a) change in degree centrality (ΔDC) and (b) clustering coefficient (ΔCC) across tasks (i.e., graph values during static subtracted from motion condition) for six functional networks of the Dosenbach atlas for healthy controls (HCs) and patients (PPV). Values above zero indicated nodes in the respective network had an AUC value during motion on average, whereas values below zero mean nodes in the network had a higher AUC value on average during the static condition. White cross indicates mean, stars indicate a significant (*p* < .05) group effect, and outliers are marked with a black cross. AUC, area under the curve; PPV, phobic postural vertigo

Subsequent *t* tests using Tukey's method revealed that the difference between groups was significant for the sensorimotor network (*t*_(167.99)_ = −3.467, *p* = .0007). PPV patients showed a significantly higher positive change, compared to HC. Conversely, HC showed a significantly higher positive change of DC in the cerebellar network (*T*_(167.99)_ = 2.389, *p* = .018). No significant group difference changes were found in the other networks for DC (Figure 4a).

For ΔCC, a significant interaction between the factor of group and network (*F*_(5,160)_ = 3.003, *p* = .013) was found. There was no significant group (*F*_(1,32)_ = 2.167, *p* = .151) or age effect (*F*_(1,32)_ = 0.928, *p* = .343). A significant main effect of network (*F*_(5,160)_ = 3.500, *p* = .005) was also found. Because of the significant interaction, post hoc *t* tests were performed. A significant difference of ΔCC between groups in the sensorimotor network was found again (*t*_(185.94)_ = −3.627, *p* = .0004). PPV patients displayed a significantly higher positive change of CC, compared to HC. No other significant group differences were found in other networks (Figure 4b).

**FIGURE 5:**
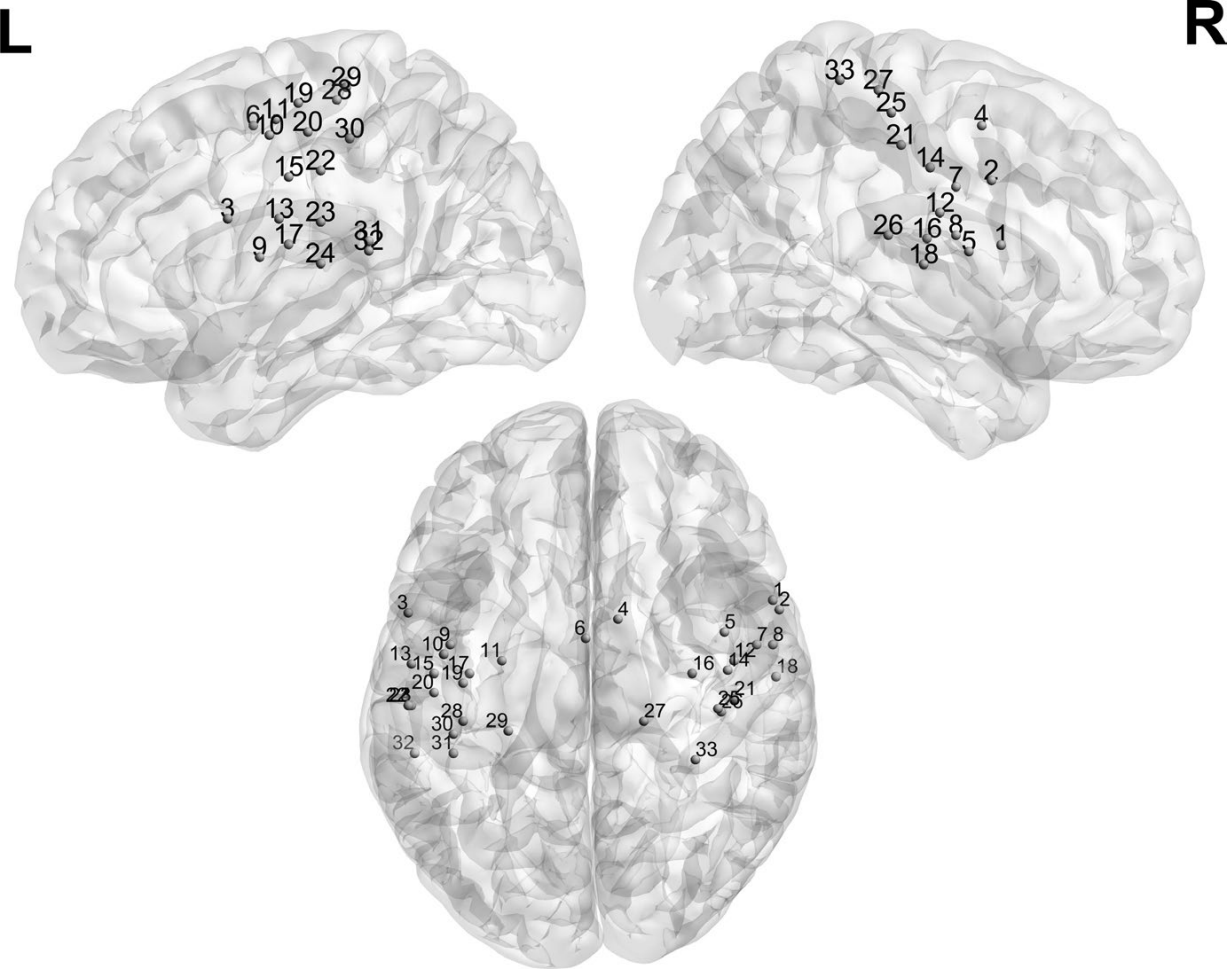
Nodes of the sensorimotor network defined according to Dosenbach (2010)

**FIGURE 6:**
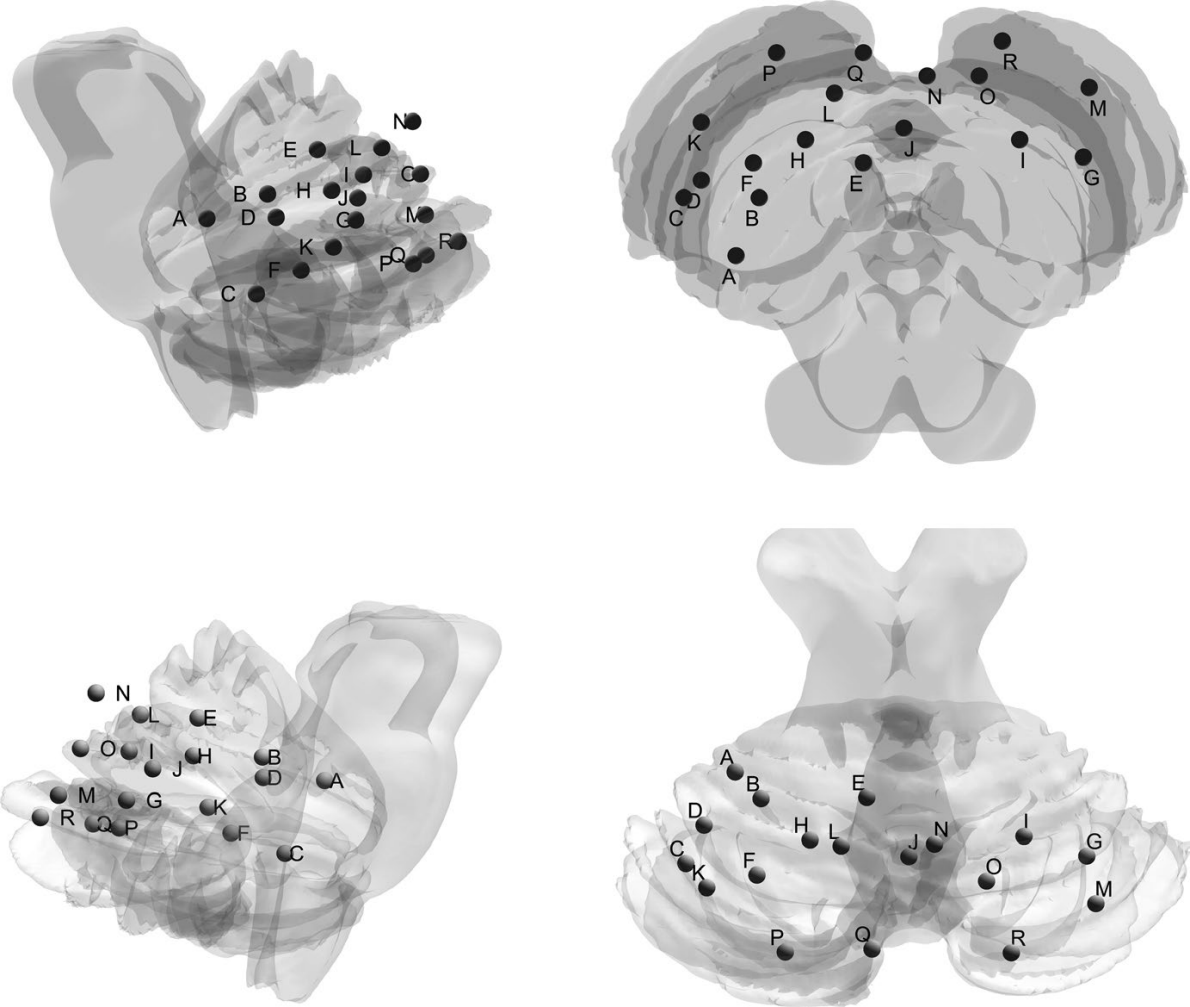
Nodes of the cerebellar network defined according to Dosenbach (2010)

The results for ΔCC were maintained when the analysis performed on the values that were normalized to random networks. However, an additional significant interaction of network and group was found in ΔECC, with post hoc *t* tests showing that PPV patients had a significantly increased ECC in the sensorimotor network compared to the HC group (*t*_(63.51)_ = −2.217, *p* = .030) (see Supplementary Information, Analysis 2 for details). An overview of the nodes from the sensorimotor and cerebellar networks can be found in Table 1 and Figure 5 and Table 2 and Figure 6, respectively.

**TABLE 1:** Coordinates and labels of nodes in the sensorimotor network (after Dosenbach et al., 2010)

Coordinates			Node	Number
58	11	14	Frontal	1
60	8	34	dFC	2
−55	7	23	vFC	3
10	5	51	Pre‐SMA	4
43	1	12	vFC	5
0	−1	52	SMA	6
53	−3	32	Frontal	7
58	−3	17	Precentral gyrus	8
−42	−3	11	Mid‐insula	9
−44	−6	49	Precentral gyrus	10
−26	−8	54	Parietal	11
46	−8	24	Precentral gyrus	12
−54	−9	23	Precentral gyrus	13
44	−11	38	Precentral gyrus	14
−47	−12	36	Parietal	15
33	−12	16	Mid‐insula	16
−36	−12	15	Mid‐insula	17
59	−13	8	Temporal	18
−38	−15	59	Parietal	19
−47	−18	50	Parietal	20
46	−20	45	Parietal	21
−55	−22	38	Parietal	22
−54	−22	22	Precentral gyrus	23
−54	−22	9	Temporal	24
41	−23	55	Parietal	25
42	−24	17	Posterior insula	26
18	−27	62	Parietal	27
−38	−27	60	Parietal	28
−24	−30	64	Parietal	29
−41	−31	48	Posterior parietal	30
−41	−37	16	Temporal	31
−53	−37	13	Temporal	32
34	−39	65	Superior parietal	33

Abbreviations: dFC, dorsal frontal cortex; SMA, supplementary motor area; vFC, ventral frontal cortex.

**TABLE 2:** Coordinates and labels of nodes in the cerebellar network (after Dosenbach et al., 2010)

Coordinates			Node	Number
−28	−44	−25	Lateral cerebellum	A
−24	−54	−21	Lateral cerebellum	B
−37	−54	−37	Inferior cerebellum	C
−34	−57	−24	Lateral cerebellum	D
−6	−60	−15	Medial cerebellum	E
−25	−60	−34	Inferior cerebellum	F
32	−61	−31	Inferior cerebellum	G
−16	−64	−21	Medial cerebellum	H
21	−64	−22	Lateral cerebellum	I
1	−66	−24	Medial cerebellum	J
−34	−67	−29	Inferior cerebellum	K
−11	−72	−14	Medial cerebellum	L
33	−73	−30	Inferior cerebellum	M
5	−75	−11	Medial cerebellum	N
14	−75	−21	Medial cerebellum	O
−21	−79	−33	Inferior cerebellum	P
−6	−79	−33	Inferior cerebellum	Q
18	−81	−33	Inferior cerebellum	R

### 3.3 Sources of differences in connectivity within cerebellar and sensorimotor network

Considering that cerebellar and sensorimotor networks showed significant network property changes between experimental condition and across groups, the question arises: What about these networks led to a change in DC? Three options were conceivable: an increase/decrease in connections (a) with nodes within the same network, (b) between nodes of the cerebellar and sensorimotor network specifically, and (c) to nodes of all the remaining networks in the brain (i.e., to the cingulo‐opercular, fronto‐parietal, default‐mode, and occipital networks).

To determine this, the same adjacency matrix values were used for the analyses described before, but the matrices were reduced in size to test each of the three options. To test for within‐network connectivity, DC for nodes of only one network (either cerebellar or sensorimotor) was calculated. To examine connectivity between the cerebellar and sensorimotor network, DC only between nodes of these networks was calculated (i.e., adjacency matrices were created containing only the correlation values of sensorimotor nodes to cerebellar nodes or vice versa). To determine the connectivity to the remaining networks, adjacency matrices containing only correlation values of either cerebellar or sensorimotor networks to nodes in the remaining networks were calculated. Other than the reduction of the adjacency matrices, the methodology was the same as described in Section 2.5 (also see Figure 1). To determine how PPV patients differed from HC, a 2 × 2 mixed‐design ANOVA with the repeated‐measure factors of connectivity type (within‐network connectivity, reciprocal connectivity, and other remaining connectivity) and the independent factor of group (HC and PPV) was performed for DC values during static and for ΔDC values.

For cerebellar connectivity during static vision, no main effect of group was found (HC and PPV patients) (*F*_(1,33)_ = 2.678, *p* = .11). Therefore, differences in cerebellar DC between groups seem not to be driven by distinct patterns in within or between connectivity (Figure A.6a). In sensorimotor connectivity during static vision, a significant effect of the factor group was found *F*_(1,33)_ = 5.68, *p* = .023). In subsequent post hoc tests, a significant effect of within‐connectivity was found with PPV showing significantly lower within‐network connectivity (*t*_(98.39)_ = 2.893, *p* = .005). Therefore, differences in DC between groups seem to be driven by connectivity changes within the somatosensory network (Figure A.6b).

When analyzing ΔDC values, again for cerebellum, no group effect was found (*F*_(1,33)_ = 1.72, *p* = .20). Again, this suggests that no distinct connectivity changes occur (Figure A.4a) in the cerebellar network. For sensorimotor connectivity, a main effect for the factor group was found (*F*(_1,33)_ = 11.786, *p* = .002). Post hoc *t* tests revealed a significant effect both in within‐sensorimotor connectivity (*t*_(98.35)_ = −2.934, *p* = .004) and in remaining connectivity to other brain networks (*t*_(98.35)_ = −3.157, *p* = .002), with PPV patients showing higher ΔDC than HC. Therefore, both within‐connectivity and connectivity to the remaining brain contributed to differences in ΔDC between groups (Figure A.7b).

## 4 DISCUSSION

The aim of the current analysis was to study the whole‐brain network properties in functional dizziness. We further wished to disentangle intrinsic network effects related to visual motion processing from network effects during static visual processing. For this, graph theory was used to characterize six functional brain networks (cingulo‐opercular, fronto‐parietal, default‐mode, sensorimotor, occipital, and cerebellar network) during periods of visual motion and interjacent periods of a static visual stimulation. Importantly, the effect of the task was regressed out from the main signal to study interaction of regions above and beyond task co‐activations. Based on previous behavioral findings (Holmberg et al., 2009; Querner et al., 2000; Wuehr et al., 2017), we expected the sensory systems and the cerebellum to show the strongest changes in network properties.

To summarize, we found that brain networks of PPV patients are connected differently (i.e., they differed in their DC) in the two conditions studied, compared to HC. During static visual stimulation, the default‐mode network as well as the cerebellar network was found to be more strongly connected in PPV. This was accompanied by a lower connectivity of the sensorimotor network. Upon visual motion stimulation, the sensorimotor network of PPV patients became significantly more connected, while the cerebellar network became less connected compared to HC. Building on the previous study by Popp et al. (2018), we also find different connectivity of cerebellum. The significant changes of network properties within the sensorimotor network during the two visual stimulation periods in PPV patients are particularly notable. We found that the sensorimotor network initially displayed decreased DC and CC during static visual stimulation, but that these measures increased to a greater extent in PPV patients during visual motion. The significant differences in DC and CC suggest changes in importance and functional segregation of the sensorimotor network, respectively. To understand these results, it is helpful to understand that sensorimotor nodes are located in, amongst others, premotor regions, the supplementary motor area, and precentral gyrus (see Table 1 and Figure 5). These regions are thought to belong to the action‐oriented motor network and are active during imagined vestibular sensation (zu Eulenburg, Müller‐Forell, & Dieterich, 2013).

The cerebellar network also had different network properties in PPV patients. The cerebellar network was connected more strongly in the static condition of PPV patients, and it did not display the same increase in DC upon motion stimulation, as is seen in HC. Aberrant cerebellar connectivity in functional dizziness has been also found during resting state, with an increase in connectivity to the thalamus (Van Ombergen et al., 2017) and a decrease in connectivity to other brain regions (Lee et al., 2018). The cerebellum is, amongst others, considered to be responsible for predicting sensory information to optimize perception (Baumann et al., 2015), displaying enhanced activity upon the absence of an expected somatosensory stimulus (Tesche & Karhu, 2000). Based on these findings, it would be interesting to investigate whether increased DC of the cerebellum during static conditions is related to a dysfunctional stimulus prediction in PPV patients. Specifically, in a state without specific motion input, increased cerebellar integration to the remaining brain network may reflect inappropriate stimulus expectations, a possible mechanism for the overpreparedness of PPV patients for motion stimuli.

The default‐mode network was found to have a higher mean DC in PPV patients during the static visual condition, when compared to HC, but no different dynamics were found between the two visual conditions. This network consists of nodes extracted from precuneus, prefrontal cortex, anterior cingulate cortex, frontal cortex, and occipital regions (see Dosenbach et al., 2010). These regions were reported to support emotional processing, self‐referential mental activity, and recollection of previous experiences (Raichle, 2015), and aberrant default‐mode resting‐state connectivity was also found in patients diagnosed with major depressive disorders (Sheline, Price, Yan, & Mintun, 2010; Whitfield‐Gabrieli & Ford, 2012). Depression as well as anxiety disorders often displays with functional dizziness (Staab et al., 2017). It would be interesting to test whether anxiety and depression are related to default‐mode network connectivity changes in PPV—since we were not specifically interested in affective disorders, this research question was, however, out of the scope of our current study. Other sensory networks did not differ in terms of their modulation between groups. The differences found in the occipital network were not statistically robust when correcting for motion, thus suggesting no direct involvement of the occipital network in PPV. This is contrary to previous findings (Lee et al., 2018; van Ombergen et al., 2017).

The presented findings are an extension of the initial analysis by Popp et al. (2018), who conducted a voxel‐based morphometry (VBM) analysis, task‐based fMRI, and task‐based functional connectivity of selected seed regions. In the latter study, structural differences between PPV patients and HC were found in cerebellum, as well as precentral gyrus and primary motor cortical areas (largely part of the sensorimotor network), but also thalamus, left supramarginal gyrus, and middle frontal gyrus. Interestingly, in the task‐based fMRI analysis only a significant increased BOLD signal in the subgenual anterior cingulate cortex was seen in PPV, hinting at more complex functional differences. Using task‐based functional connectivity of six selected seeds (based on the findings of the VBM analysis), differences in the cerebellum and precentral gyrus were found amongst others (Popp et al., 2018). In the current study, we expanded on these findings using a functional network analysis across the whole brain (rather than extracting seeds) and took advantage of the different task episodes (static and motion). Indeed, we also found an involvement of premotor areas and cerebellar networks, particularly upon visual motion stimulation.

Taken together, we hypothesize that network changes found in PPV patients can be connected to the mechanistic models of sensory efference copy (von Holst & Mittelstaedt, 1950) or the related Bayesian modeling approach (Henningsen et al., 2018; Petzschner, Weber, Gard, & Stephan, 2017). The first model explains the tendency of vertigo patients to perceive involuntary bodily fluctuations and individual head movements as a disturbing external acceleration by a transient uncoupling of efference and efference copy, leading to a mismatch between anticipated and actual motion (Brandt, 1996; Henningsen et al., 2018; Petzschner et al., 2017). In the latter model, perception or beliefs are considered to be an inferred process. Here, abnormal signaling or computation of priors, prediction errors, or precision ratios leads to functional somatic syndromes such as PPV (see Petzschner et al., 2017 for more details). Connecting this to the present findings, we suggest that in the absence of visual motion stimulation, networks associated with stimulus expectations (cerebellar network) and increased focus on internal processes (default‐mode network) are overprioritized in PPV. Conversely, the sensorimotor network is less important in PPV during static visual input. Upon visual motion, regions involved with action‐oriented evaluation of sensory stimuli become overprioritized upon sensory input in patients. To test the hypothesis that the differences in network dynamics are related to differences in stimulus expectation and evaluation, it would be necessary to include behavioral measures which test for dysfunctional interpretation of sensory input and to connect them to changes in connectivity measured by means of fMRI.

Overall, in the present study we took a whole‐brain, network‐level approach to characterize changes in the brain of PPV patients when compared to HCs. Therefore, we did not aim to reach any conclusions regarding how individual nodes/brain regions are implicated. We restricted our graph theoretical approach to three simple and widely used measures (DC, CC, and ECC) to investigate importance, functional segregation, and functional integration of the networks. We did not find any differences in ECC in any of our measurements.

A limitation of our study is that eye movements were monitored but not recorded. Although relevant ocular motor phenomena or neuroophthalmological pathologies have already been excluded in the diagnosis process, we cannot completely exclude that subtle differences in ocular motor behavior explain the differences in functional connectivity. In future studies, it would be interesting to record and analyze eye movements during such a visual motion paradigm to determine potential influences on connectivity. Another limitation of the study is that the presented findings may not be unique to PPV. Firstly, due to the comorbidity of PPV with depression and anxiety the network‐level changes found in PPV may not be specific to functional dizziness, but rather depression or anxiety in general. Future studies should include populations of individuals with similar levels of trait anxiety and depression (but without dizziness symptoms) to evaluate specificity of the described results. Furthermore, previous studies suggested that visual dependency is related to chronic functional dizziness symptoms (Cousins et al., 2014, 2017). In future, recording visual dependency in a similar manner would be useful to determine the relation of our reported functional brain changes to such visual motion sensitivity.

## 5 CONCLUSIONS

Distinct changes in functional brain networks in PPV patients during static visual stimulation were found in nodes of the sensorimotor network, the cerebellar network, and the default‐mode network. Upon visual motion, nodes in the sensorimotor network become more connected in PPV, whereas cerebellar nodes become more connected in HC. We hypothesize that the underlying network differences may be related to dysfunctional stimulus expectations and suggest combining functional brain network analysis with psychophysical approaches in PPV patients using Bayesian modeling.

## CONFLICT OF INTEREST

All authors declared no conflict of interest.

## AUTHOR CONTRIBUTION

JH conceived the analysis, analyzed and interpreted the data, wrote the manuscript, and created the figures. VLF provided support with data analysis, and drafted and reviewed the manuscript and figures. PP acquired the data. PzE conceived the analysis, interpreted the data, and drafted and reviewed the manuscript and figures. MD conceived and designed the study, and drafted and reviewed the manuscript and figures.

## Supporting information

Figure A1

Figure A2

Figure A3

Figure A4

Figure A5

Figure A6

Figure A7

Figure B1

Figure B2

Figure B3

Figure B4

## Data Availability

The data that support the findings of this study are available from the corresponding author upon reasonable request.
