# Supplementary figures and images for: Network changes in patients with phobic postural vertigo
Source: Brain Behav. 2020 Apr 18;10(6):e01622. doi: 10.1002/brb3.1622 (PMC7303402; doi:10.1002/brb3.1622)

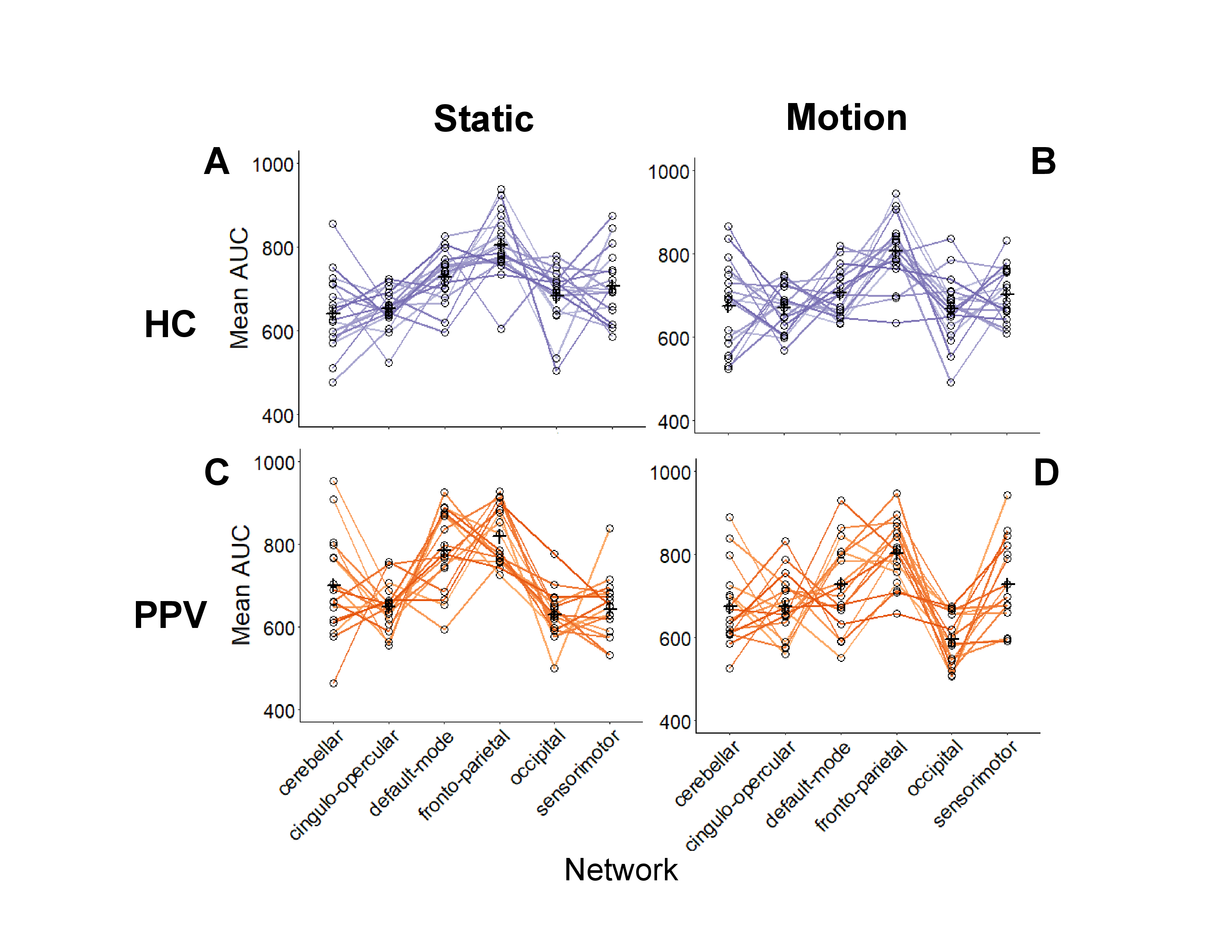

Supplement: Supplementary file 1 — Figure A1 [file BRB3-10-e01622-s001.tiff]

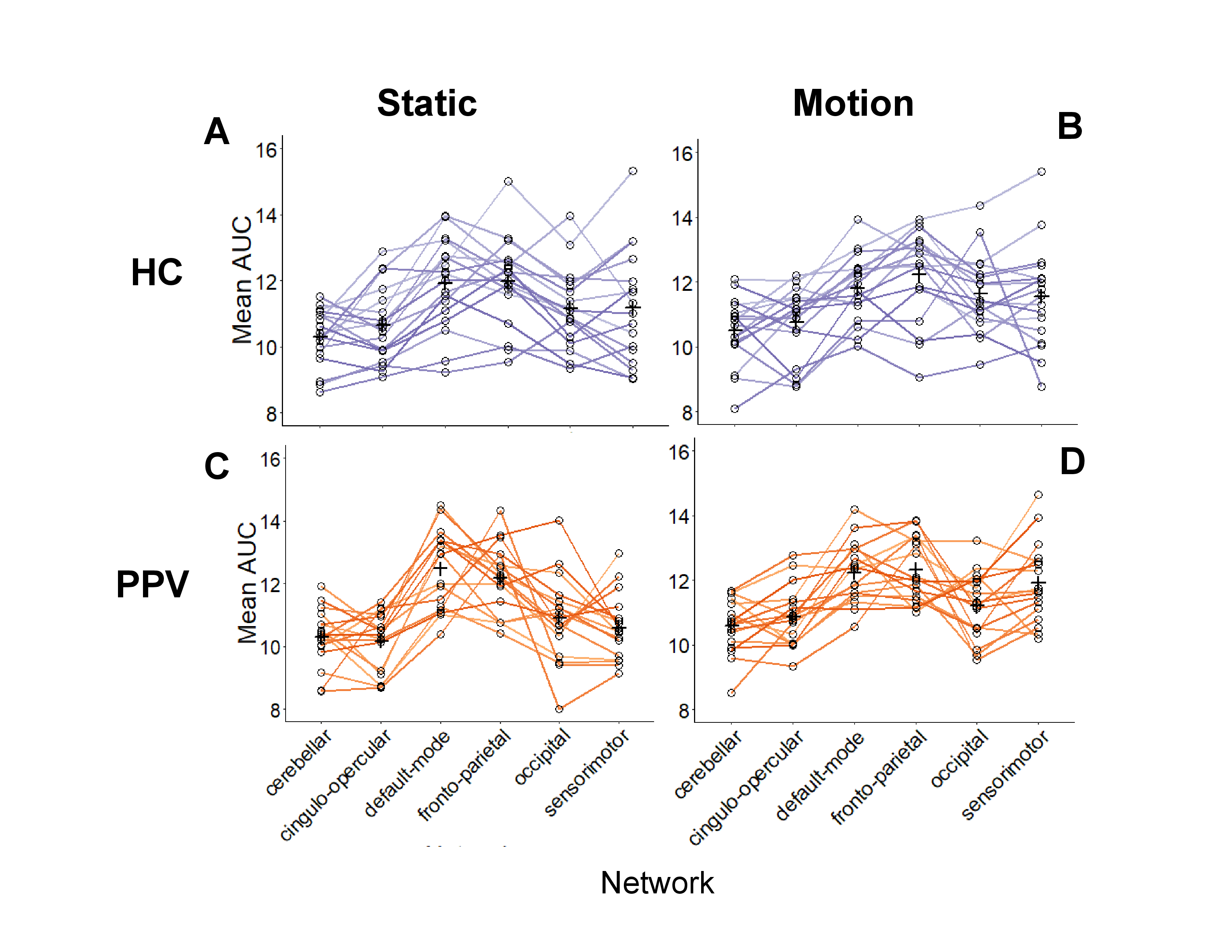

Supplement: Supplementary file 2 — Figure A2 [file BRB3-10-e01622-s004.tiff]

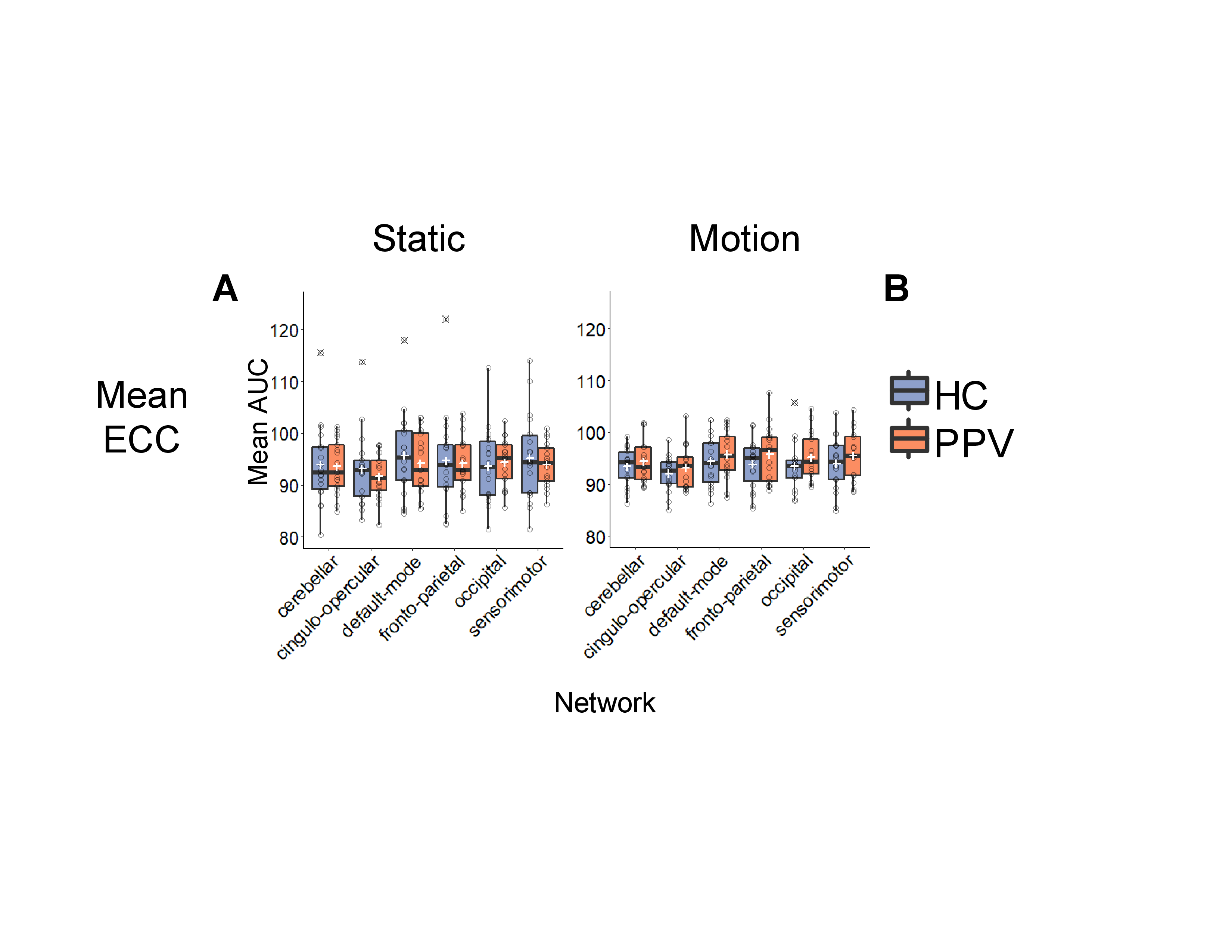

Supplement: Supplementary file 3 — Figure A3 [file BRB3-10-e01622-s005.tiff]

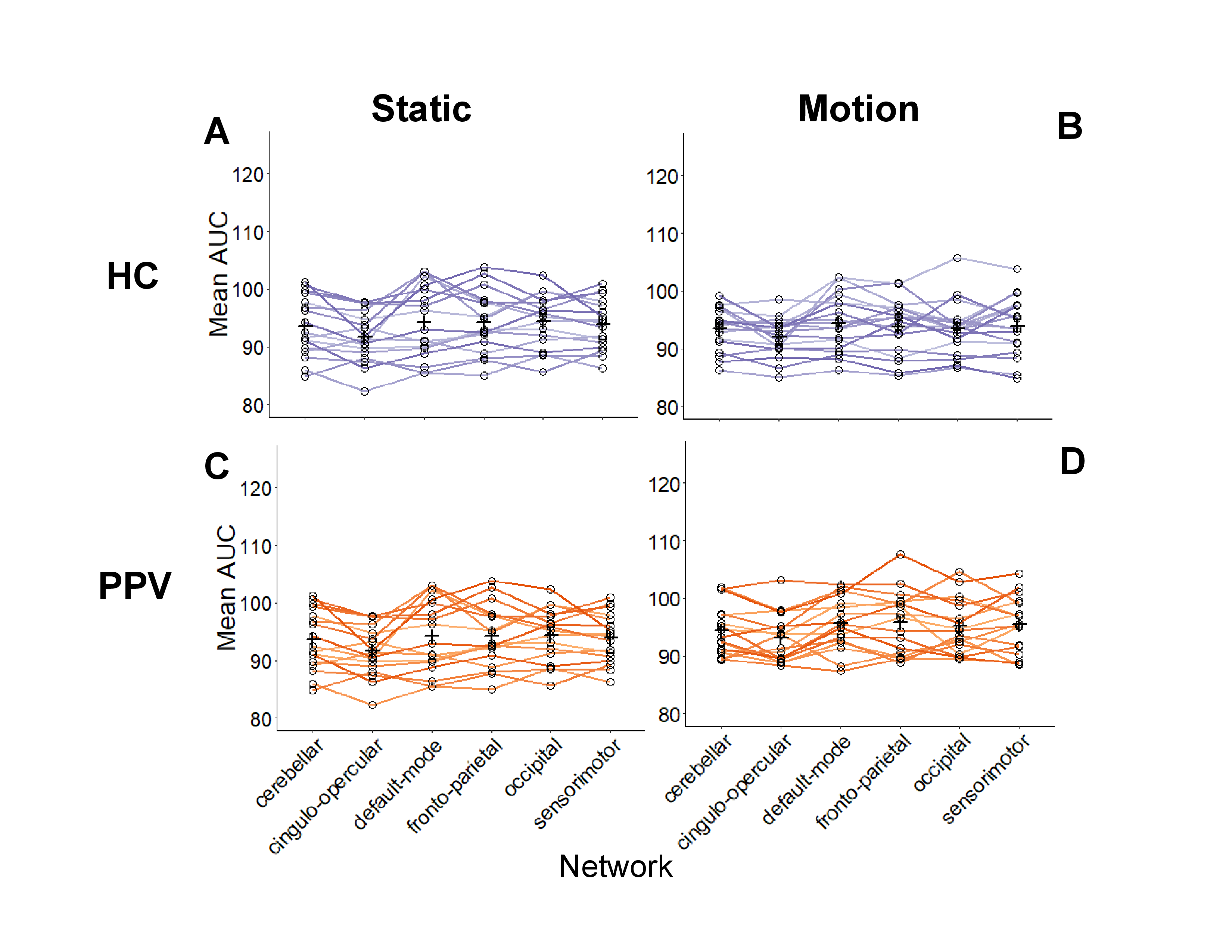

Supplement: Supplementary file 4 — Figure A4 [file BRB3-10-e01622-s006.tiff]

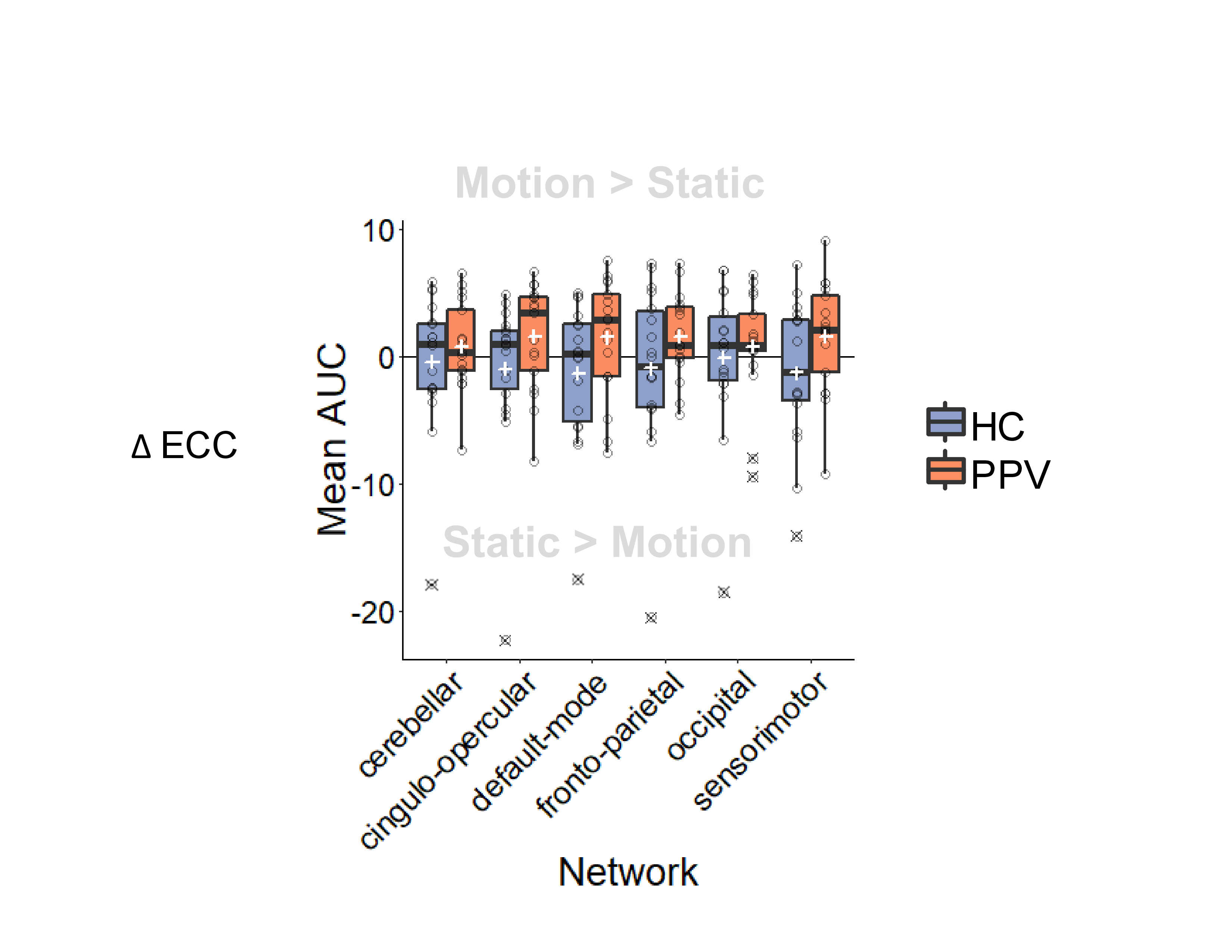

Supplement: Supplementary file 5 — Figure A5 [file BRB3-10-e01622-s007.tiff]

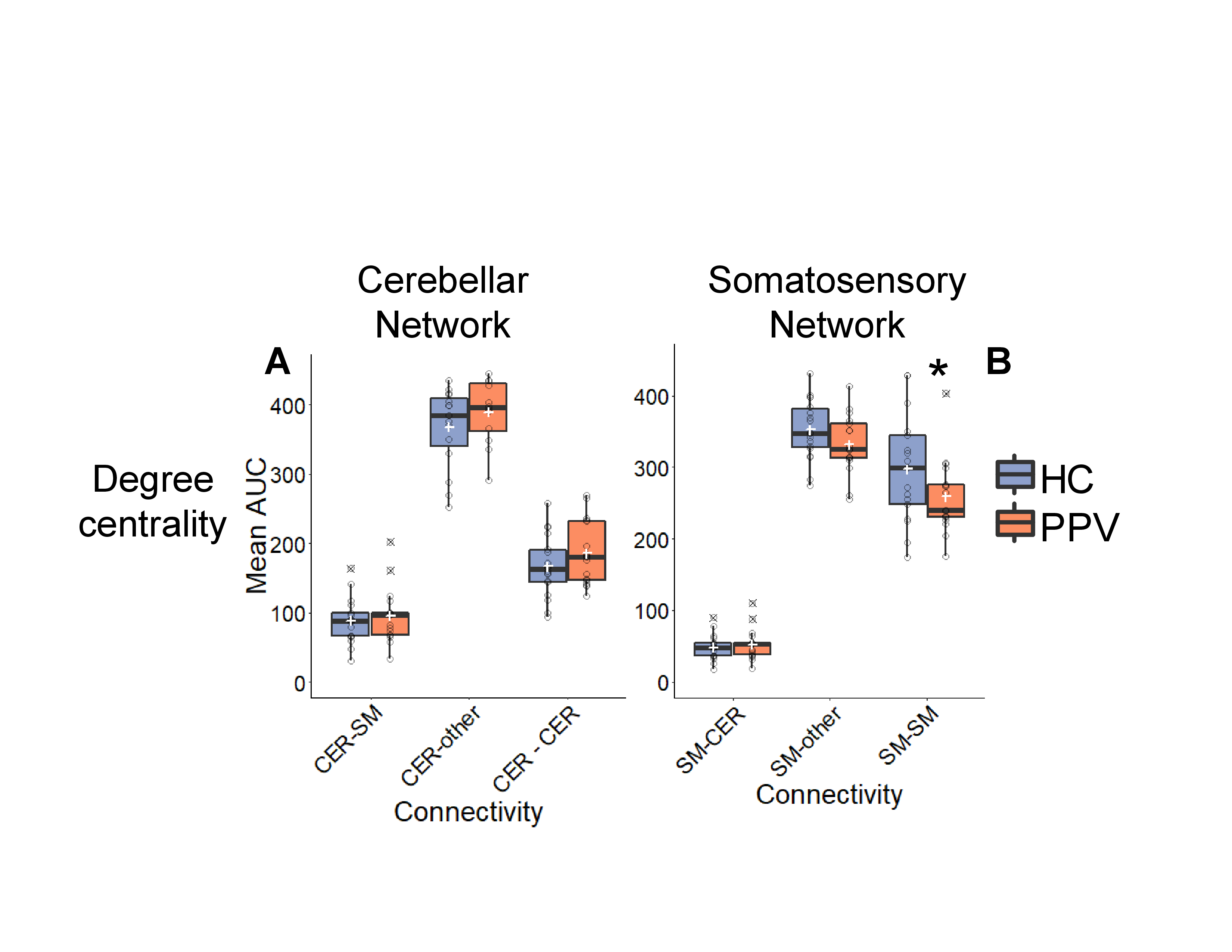

Supplement: Supplementary file 6 — Figure A6 [file BRB3-10-e01622-s008.tiff]

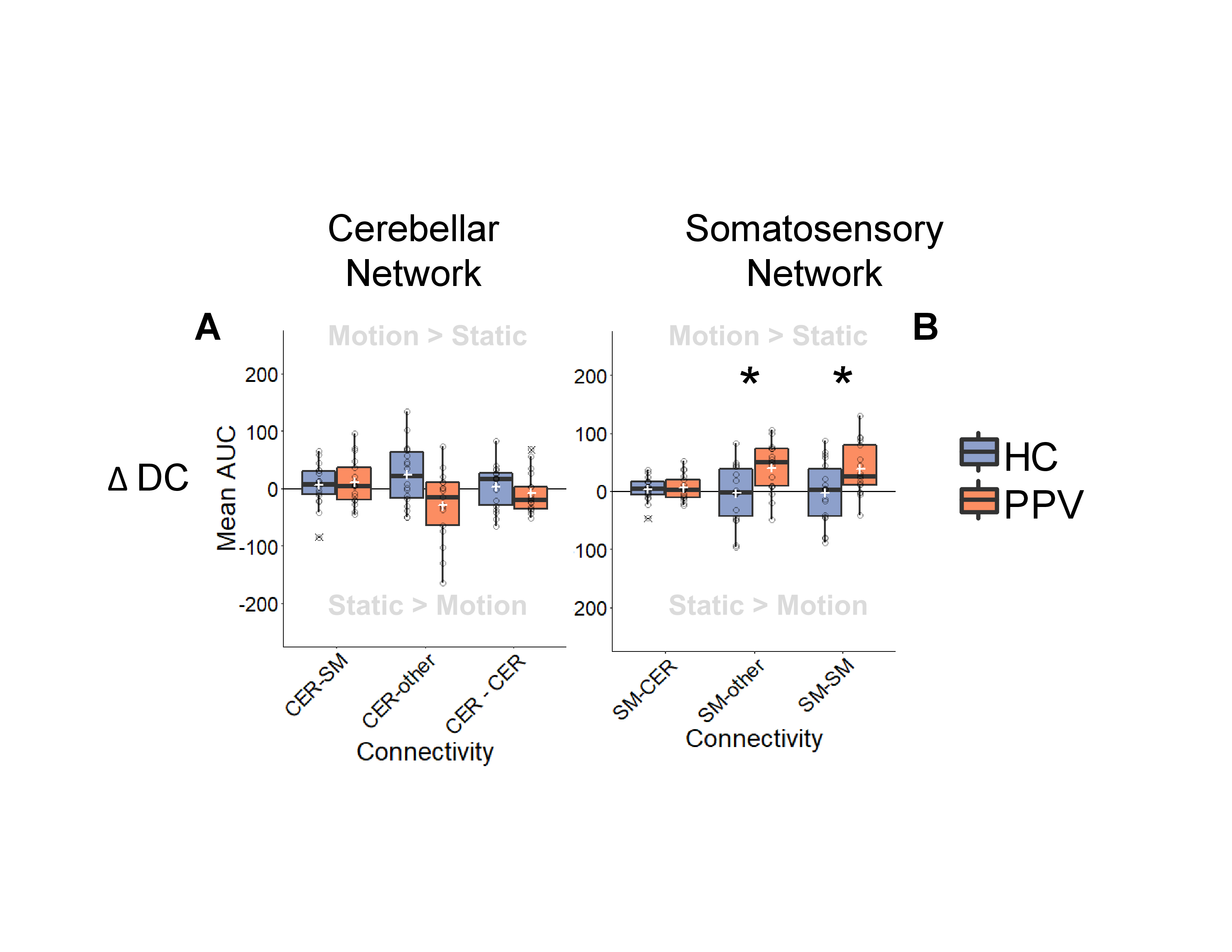

Supplement: Supplementary file 7 — Figure A7 [file BRB3-10-e01622-s009.tiff]

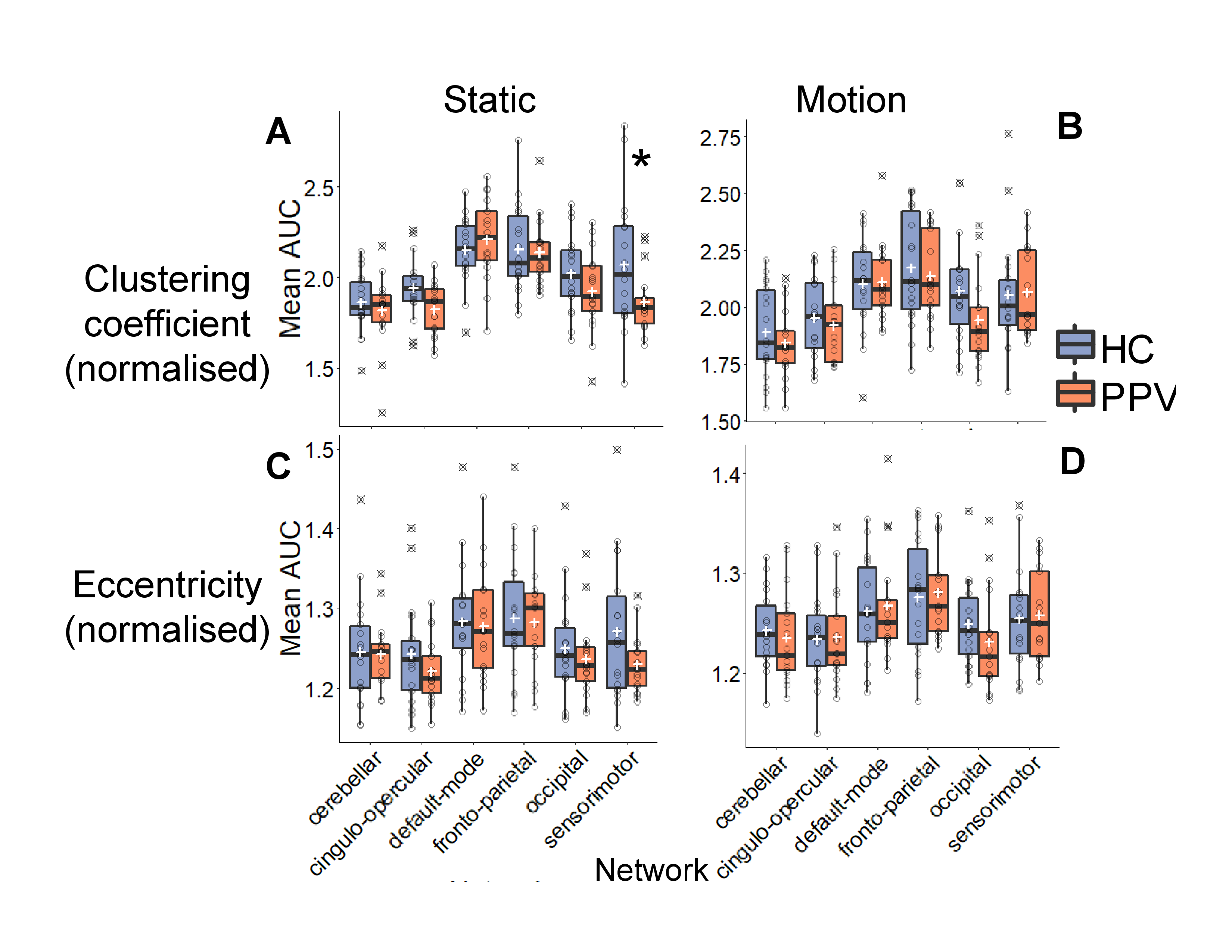

Supplement: Supplementary file 8 — Figure B1 [file BRB3-10-e01622-s010.tiff]

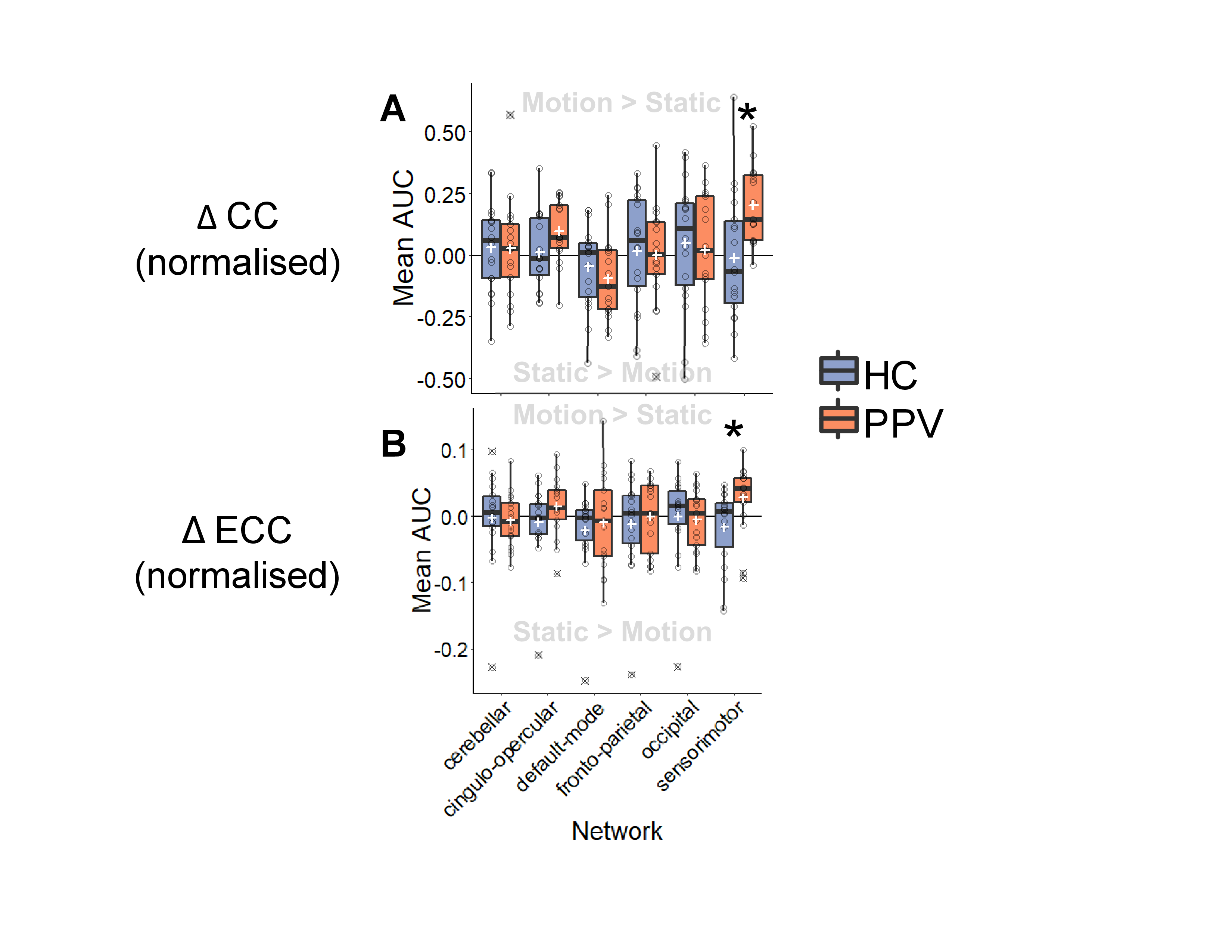

Supplement: Supplementary file 9 — Figure B2 [file BRB3-10-e01622-s011.tiff]

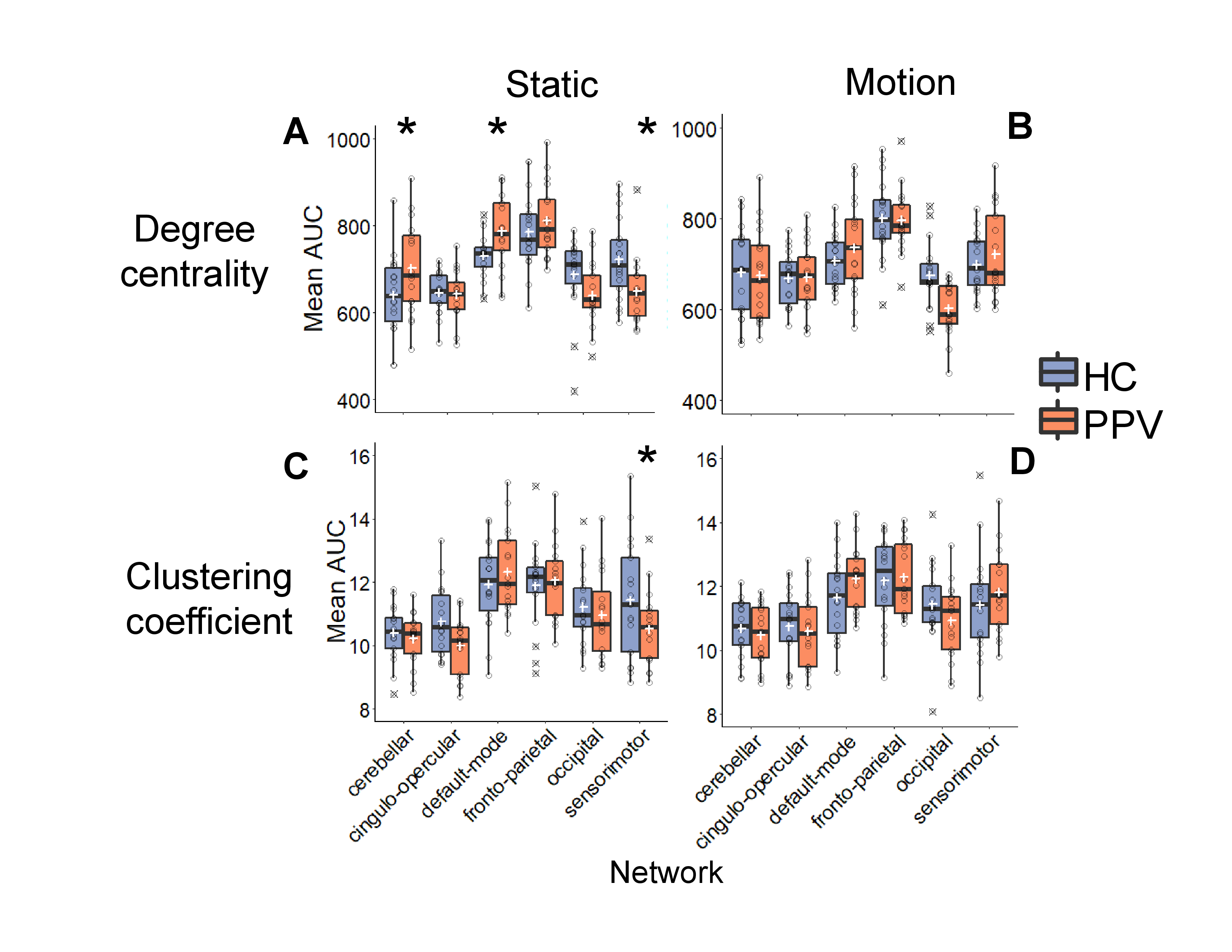

Supplement: Supplementary file 10 — Figure B3 [file BRB3-10-e01622-s002.tiff]

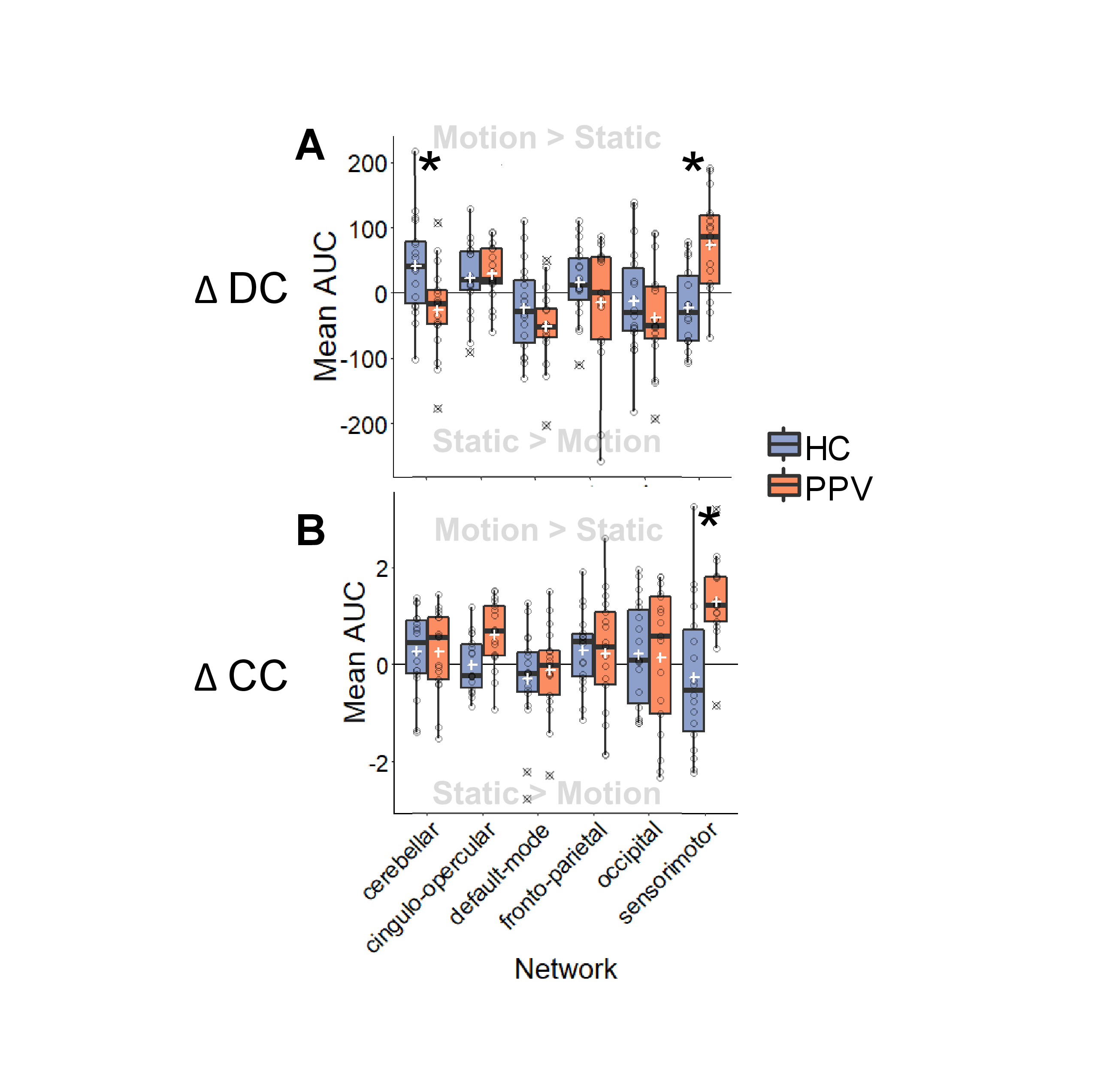

Supplement: Supplementary file 11 — Figure B4 [file BRB3-10-e01622-s003.tiff]
